# Differential nasopharyngeal microbiota composition in children according to respiratory health status

**DOI:** 10.1099/mgen.0.000661

**Published:** 2021-10-26

**Authors:** Desiree Henares, Pedro Brotons, Mariona F. de Sevilla, Ana Fernandez-Lopez, Susanna Hernandez-Bou, Amaresh Perez-Argüello, Alex Mira, Carmen Muñoz-Almagro, Raul Cabrera-Rubio

**Affiliations:** ^1^​ Institut de Recerca Sant Joan de Deu, Hospital Sant Joan de Deu, Barcelona, Spain; ^2^​ CIBER Center for Epidemiology and Public Health (CIBERESP), Instituto de Salud Carlos III, Madrid, Spain; ^3^​ School of Medicine, Universitat Internacional de Catalunya, Barcelona, Spain; ^4^​ Pediatric Department, Hospital Sant Joan de Deu, Barcelona, Spain; ^5^​ Pediatric Emergency Department, Hospital Sant Joan de Deu, Barcelona, Spain; ^6^​ Department of Health and Genomics, Center for Advanced Research in Public Health, FISABIO, Valencia, Spain; ^7^​ Teagasc Food Research Centre (TEAGASC), Moorepark, Fermoy, Cork, Ireland; ^8^​ APC Microbiome Institute, University College Cork, County Cork, Ireland

**Keywords:** children, *Dolosigranulum*, IPD, microbiota-based diagnostics, nasopharyngeal microbiota, viral URTIs

## Abstract

Acute respiratory infections (ARIs) constitute one of the leading causes of antibiotic administration, hospitalization and death among children <5 years old. The upper respiratory tract microbiota has been suggested to explain differential susceptibility to ARIs and modulate ARI severity. The aim of the present study was to investigate the relation of nasopharyngeal microbiota and other microbiological parameters with respiratory health and disease, and to assess nasopharyngeal microbiota diagnostic utility for discriminating between different respiratory health statuses. We conducted a prospective case–control study at Hospital Sant Joan de Deu (Barcelona, Spain) from 2014 to 2018. This study included three groups of children <18 years with gradual decrease of ARI severity: cases with invasive pneumococcal disease (IPD) (representative of lower respiratory tract infections and systemic infections), symptomatic controls with mild viral upper respiratory tract infections (URTI), and healthy/asymptomatic controls according to an approximate case–control ratio 1:2. Nasopharyngeal samples were collected from participants for detection, quantification and serotyping of pneumococcal DNA, viral DNA/RNA detection and 16S rRNA gene sequencing. Microbiological parameters were included on case–control classification models. A total of 140 subjects were recruited (IPD=27, URTI=48, healthy/asymptomatic control=65). Children’s nasopharyngeal microbiota composition varied according to respiratory health status and infection severity. The IPD group was characterized by overrepresentation of *Streptococcus pneumoniae *, higher frequency of invasive pneumococcal serotypes, increased rate of viral infection and underrepresentation of potential protective bacterial species such as *Dolosigranulum pigrum* and *Moraxella lincolnii *. Microbiota-based classification models differentiated cases from controls with moderately high accuracy. These results demonstrate the close relationship existing between a child’s nasopharyngeal microbiota and respiratory health, and provide initial evidence of the potential of microbiota-based diagnostics for differential diagnosis of severe ARIs using non-invasive samples.

## Data Summary

Raw sequence files have been deposited in the European Nucleotide Archive (ENA) at EMBL-EBI under accession number PRJEB41741. The metadata file, the nucleotide sequences of the OTUs (operational taxonomic units), the OTU table (clean), the corresponding taxonomic classifications, the phylogenetic tree, and the Appendices S1, and S2 have been deposited in the Figshare repository: https://doi.org/10.6084/m9.figshare.13280435.v3 [1]. The rest of data can be found in Figs S1–S8 and Tables S1–S5, available with the online version of this article.

**Impact Statement**

Acute respiratory infections (ARIs) are a leading cause of children’s morbidity/mortality. Common pathogens causing ARIs are frequent colonizers of healthy children’s nasopharynx. The surrounding micro-environment has been suggested to play a role in the transition of these pathogens to ARIs and modulate its severity. Our case–control study demonstrated a close relationship between a child’s nasopharyngeal microbiota and respiratory health status by identifying specific microbiota profiles and species associated with health and/or ARI states. Microbiota-based classification models enabled accurate differentiation of severe ARIs requiring hospitalization. These results can help to improve preventive strategies and non-invasive diagnosis of severe ARIs.

## Introduction

Acute respiratory infections (ARIs) constitute the second highest cause of mortality among children under 5 years old, accounting for approximately 15% of all deaths in this age range [2, 3]. ARIs are also an important part of the morbidity and constitute one of the main reasons for antibiotic administration in children [4]. Common bacterial pathobionts causing ARIs, such as *Streptococcus pneumoniae *, *Haemophilus influenzae* and *Staphylococcus aureus *, are frequent colonizers of healthy children’s nasopharynx [5], but also an important cause of serious disease [6]. In particular, *S. pneumoniae* nasopharyngeal carriage rates can be as high as 95% among children <3 years old [6]. However, this pathobiont may invade sterile tissues, producing invasive pneumococcal disease (IPD) with a wide range of serious clinical manifestations, such as pneumonia, sepsis and meningitis [6]. The transition processes from balanced colonization states to IPD are not completely understood. Although carriage of especially invasive serotypes may play a role [7], increased recognition is being paid to the consideration of respiratory infections as the outcome of an imbalanced interplay between environmental and life-style determinants, and host and microbial factors. Among the latter, the commensal microbiota of the upper respiratory tract (URT) has been suggested to influence differential susceptibility to and severity of IPD [5].

The respiratory microbiome is composed of an intricate network of bacteria, viruses, fungi, bacteriophages, archaea and eukaryotes that colonize the mucosal surfaces of the respiratory tract and continuously interact with each other and with the host and the environment [5]. This colonization process begins early at birth, with very unstable initial communities that are heavily influenced by an infant’s environmental exposures. Through the first years of life, the respiratory microbiome undergoes continuous changes until achieving an adult-like stabilized structure [8]. Despite high variability of the microbiome in early life, the initial colonization by keystone pathogens has been proposed to be determinant on the future health of the infant and even on that of the adult [9]. Particularly, a recent study showed that nasopharyngeal microbiota profiles characterized by *Corynebacterium* and *Dolosigranulum* early in life and *Moraxella* at 4–6 months were associated with lower rates of respiratory events in the subsequent months [10]. In addition, vaginal delivery, breastfeeding and vaccination strategies have been reported to drive beneficial effects on respiratory health through their influence in modulation of commensal microbiota from the URT at initial ages [11–13]. The consistency and importance of these findings are under discussion [14–16].

Antibiotics and viral infections have been described as frequent and important events disturbing normal development of healthy infant respiratory microbiota. A reduction of the commensal microbiota, such as *Corynebacterium* or *Dolosigranulum *, while displaying enrichment of bacterial pathogens, has been observed associated with antibiotic consumption or during upper respiratory tract infections (URTIs), including those of viral aetiology [8, 14, 17–19]. Moreover, recent literature concluded that influenza A viral infections perturb the core URT microbiome via direct and indirect processes that could facilitate bacterial co-infections, suggesting that viral–bacterial interactions in the ecological niche may be of extreme relevance to the pathogenesis of ARIs [20]. Of note, the low accuracy of current microbiological methods to distinguish viral from bacterial ARIs is a major cause of unnecessary use of antibiotics [21]. Altogether, an imbalanced state and dysbiosis of the URT microbiota may reduce its capability to perform vital functions, including pathogen colonization resistance, and containment of pathogenic overgrowth and invasion, tipping the balance towards progression to URTIs and lower respiratory tract infections (LRTIs), such as pneumonia, as well as to systemic infections [22–25].

The primary objective of this study was to investigate the relation of children’s nasopharyngeal microbiota with ARI across three respiratory health statuses: IPD (a condition representative of LRTIs and systemic infection), self-limiting viral respiratory infections (a condition representative of URTIs), and respiratory health/asymptomatic colonization. A secondary objective was to assess nasopharyngeal microbiota diagnostic utility for differentiating between these different respiratory states. Our underlying hypotheses were that specific microbiota compositions are correlated with different respiratory infectious disease and health states, and have diagnostic value to differentiate one from another. In addition, this study assessed the influence of different environmental variables on microbiota composition.

## Methods

### Study design and setting

This was a prospective case–control study conducted at Sant Joan de Deu Barcelona Children’s Hospital (HSJD), a reference university medical centre located in Barcelona, Spain, during the period from January 2014 to December 2018. The study setting annually manages 22% of the total paediatric hospitalizations across Catalonia, a region with a paediatric population of around 1 500 000 subjects. Three groups of children were selected for the study: (i) a case group of inpatients with IPD; (ii) a control group of outpatients with microbiologically confirmed symptomatic viral URTIs; (iii) and a control group of healthy or asymptomatic outpatients.

### Case and control definitions

Cases were patients less than 18 years of age admitted to HSJD with clinical suspicion of IPD and microbiological confirmation by isolation of *S. pneumoniae* and/or DNA detection of *S. pneumoniae* in any normally sterile body fluid. Cases were frequency-paired by age, sex and seasonality with controls. Controls were symptomatic children attending the Emergency Department of HSJD and being discharged to home with clinical and microbiological diagnosis of viral URTI (approximate case–control ratio 1:2), as well as healthy/asymptomatic children attending HSJD for a routine well-child check-up or for a blood extraction before minor surgery (such as circumcisions, correction of bone fractures, removal of skin lesions, hernia repairs, etc.) (approximate case–control ratio 1:2). Clinicians evaluated and confirmed the healthy/asymptomatic state of these controls.

Inclusion criteria for cases and controls were: (i) meeting the case/control definition described above, (ii) signed informed consent by the parents or legal guardians, (iii) not belonging to a previously defined clinical risk group for developing IPD, and (iv) no antibiotic exposure before nasopharyngeal sample collection for healthy/URTI controls or no antibiotic exposure/antibiotic exposure ≤24 h for IPD cases [8, 16, 26].

Clinical risk factors for developing IPD include asplenia or spleen dysfunction, chronic lower respiratory tract diseases, respiratory conditions caused by aspiration, diabetes, immunosuppression (due to disease or treatment), exposure to a high dose of systemic steroids, cochlear implants, cerebrospinal fluid leaks, and chronic heart, kidney and liver diseases [27].

### Data collection

A series of relevant epidemiological, clinical and microbiological data were recorded from each participant. Epidemiological variables included delivery mode, ethnicity, maternal breastfeeding duration according to World Health Organization (WHO) recommendations (duration ≥6 months) and pneumococcal vaccination status [categorized into non-vaccinated children or children with ≥1 dose of 7, 10 or 13-valent pneumococcal conjugate vaccines (PCVs)]. Clinical diagnosis, length of hospital stay, admission to the Paediatric Intensive Care Unit (PICU), length of PICU stay and patient evolution were collected from cases with IPD. Among microbiological variables, we determined for all participants: respiratory viral species present in the nasopharynx, nasopharyngeal bacterial microbiota, pneumococcal colonization status, pneumococcal nasopharyngeal load, invasiveness disease potential of nasopharyngeal pneumococcal serotypes and their inclusion in PCV13. Serotypes 1, 3, 4, 5, 7F, 8, 9A, 9V, 12F, 14, 18C and 19A were considered as high invasive disease potential serotypes according to previous literature [28–30]. A detailed list and description of variables collected is included in the metadata file (https://doi.org/10.6084/m9.figshare.13280435.v3).

### Sample collection

Invasive samples (blood, pleural fluid and cerebrospinal fluid) were collected from patients with clinical suspicion of IPD for microbiological diagnosis. Nasopharyngeal aspirates (NPAs) were collected from all cases and controls included in the study. Nasopharyngeal secretions were aspirated using a catheter connected to a vacuum source. The mucus collected within the catheter was then eluted by the aspiration of 1.0 ml sterile PBS). The sample was aliquoted and conserved at −80 °C until further microbial analyses.

### Laboratory analyses

#### DNA/RNA extraction procedures

Bacterial DNA was extracted from *S. pneumoniae *-positive invasive samples and NPAs by the automated system NucliSENS easyMag (bioMérieux) from a total sample volume of 400 µl into a final elution volume of 110 µl for pneumococcal detection/quantification/serotyping. A NPA volume of 400 µl eluted in 25 µl was utilized for microbiota analyses. The NucliSENS easyMag is an automated system for total nucleic acid extraction that utilizes magnetic silica particles. No additional treatments were performed before nucleic acid extraction for microbiota analyses. Nucleic acids were extracted with the MagNA Pure Compact total nucleic acid isolation kit I (Hoffmann-La Roche) from an initial sample volume of 200 µl into a final elution volume of 100 µl for viral DNA/RNA detection.

#### Pneumococcal detection, quantification and serotyping

A duplex real-time PCR targeting the *lytA* gene and *RNase P* (single-copy gene of the human genome used as an amplification control) was used for pneumococcal DNA detection/quantification in invasive samples and NPAs [31]. Primers and probes were those recommended by the Centers for Disease Control and Prevention (CDC). Blood agar plates (Columbia agar supplemented with 5% sheep blood; bioMérieux) were utilized for the isolation of pneumococcal strains directly from invasive samples or from positive blood cultures. Standard microbiological tests were performed for *S. pneumoniae* identification, including the study of sensitivity by optochin tests [31]. Culture, PCR or both microbiological methods were routinely performed on invasive samples from patients with clinical suspicion of IPD according to paediatrician’s criteria. All invasive and colonizing *S. pneumoniae* detected were further serotyped (Appendix S1: https://doi.org/10.6084/m9.figshare.13280435.v3).

#### Viral RNA/DNA detection

All NPAs were tested using the multiplex real-time PCR Anyplex II RV16 detection kit (Seegene). This system is able to detect and differentiate DNA/RNA from 15 of the most frequent human respiratory viruses (metadata file: https://doi.org/10.6084/m9.figshare.13280435.v3).

#### Bacterial 16S rRNA gene sequencing

PCR amplification of the V3-V4 region from the 16S rRNA gene generated amplicons of approximately 465 bp in length. These amplicons were pooled and sequenced with the MiSeq platform (Illumina). Samples were sequenced in four different runs and 16 negative controls were included. A complete description of the sequencing procedure and negative controls included is specified in Appendix S1 (https://doi.org/10.6084/m9.figshare.13280435.v3).

### Bioinformatics procedures

Filtering of raw reads by sequence quality and length was performed with PRINSEQ-Lite v0.20.4 [32]. flash v1.2.11 was utilized for merging paired-end reads [33]. Only those samples with ≥30 000 high-quality sequences were included for subsequent analyses. Sequences were dereplicated with Vsearch v2.13.3 and clustered into OTUs (operational taxonomic units) at 97% of identity with the uparse-algorithm-based approach from Usearch [34]. Vsearch removed chimerical OTUs applying a reference-based approach with the Gold database from the Broad Microbiome Utilities version microbiomeutil-r20110519 (https://sourceforge.net/projects/microbiomeutil/files/). The RDP Classifier v.2.12 [35] taxonomically assigned OTUs up to genus level with a minimum confidence threshold of 80%, while spingo assigned OTUs at the species level with a confidence value >75% [36]. The phylogenetic tree was reconstructed using PyNAST v0.1 [37] and Qiime 1 scripts [38] (filter_alignment.py and make_phylogeny.py with the fasttree method). Negative controls were evaluated for removal of contaminant OTUs: an abundance ratio that considers prevalence and abundance of OTUs in negative controls and biological samples was applied to the data. Additionally, the Decontam package was used for stricter contaminant elimination [39]. For alpha-diversity analyses, the count table was rarefied to 30 000 sequences per sample. For the rest of the analyses [beta-diversity, differential abundance testing and random forest (RF) analyses], the unrarefied count table was filtered by keeping OTUs with a relative abundance >0.1% in at least 10% of samples. This cut-off was applied with the objective of performing the analyses based on representative taxa, since low count and low prevalent sequences tend to represent sequencing artefacts, contaminants or transient micro-organisms without a real biological role in the studied niche [40, 41], and to assure that the taxa under consideration would be meaningful for class separation [42]. Data normalization was conducted by log-transformation of the unrarefied and filtered OTU table using log_10_(*x*+1) prior to beta-diversity and RF analyses. A relative abundance table was the input required for differential abundance testing using Linear Discriminant analysis Effect Size (LEfSe). Complete descriptions of the bioinformatics procedures are specified in Appendices S1 and S2, including a detailed explanation of OTU table clean-up steps (https://doi.org/10.6084/m9.figshare.13280435.v3).

### Statistical analyses

All statistical analyses were performed with R version 3.6.0 [43]. Normality of the data was evaluated with Shapiro–Wilk test. Continuous variables were described as mean and standard deviation (sd) or median and inter-quartile range (IQR) in the case of parametric and non-parametric variables, respectively. Significance of continuous and normally distributed data were tested by ANOVA for overall group comparisons and Tukey HSD when looking for specific mean differences between groups. In the case of non-parametric data, Kruskal–Wallis and pairwise Wilcoxon tests were performed. When analysing categorical data, significance was established through Chi-square test or Fisher’s exact test if ≥25 % of cells presented expected frequencies ≤5. Pairwise Chi-square or pairwise Fisher’s exact test were used for specific data distribution differences between groups.

Differences in species richness and diversity according to respiratory infectious disease and health states were assessed by testing the significance of Chao1 and Shannon indexes using Kruskall–Wallis and pairwise Wilcoxon tests. Overall differences in microbiota composition between states were assessed by PERmutational Multivariate ANalysis Of VAriance (PERMANOVA) from Vegan (Adonis 2 function) [44] using a Bray–Curtis dissimilarity measure. Specific differences between groups were assessed by *post hoc* comparisons with Adonis pairwise comparisons. PERMANOVA models and Adonis pairwise comparisons were repeated with Jaccard and weighted Unifrac distance matrices and visualized with principal coordinate analyses (PCoAs). With the objective of identifying other sources influencing nasopharyngeal microbiota structure, the significance of Chao1, Shannon and PERMANOVA models were also tested according to other described environmental variables relevant for microbiota composition and diversity (age, gender, breastfeeding duration ≥6 months, pneumococcal vaccination and viral infection) [8, 45, 46]. The effect of sequencing run was also evaluated in alpha- and beta-diversity analyses in order to control for the possible confounding effect of differential sequencing depth among runs. Additionally, a canonical correspondence analysis (CCA) triplot was constructed showing the relation between nasopharyngeal samples, bacterial genera detected and the significant variables influencing beta-diversity as determined by PERMANOVA models. LEfSe was performed in order to discover specific bacterial biomarkers associated with health and disease states, as well as with environmental variables [47]. Adopting a stringent criterion, bacterial taxa were considered relevant if presenting a linear discriminant analysis (LDA) score ≥3.0. Bacterial interrelations were evaluated by calculating Spearman’s correlations between dominant genera of nasopharyngeal microbiota. RF was used for building case–control classification models including all microbiological variables assessed in nasopharyngeal samples as a machine-learning approach for distinguishing cases from controls. Three different RF models were constructed for classification of: (i) IPD cases versus viral URTI controls, (ii) IPD cases versus healthy controls, and (iii) IPD cases versus all controls. RF performance was evaluated by the area under the curve (AUC) parameter. The importance of the variables to the performance of each RF model was measured by mean decrease in Gini index. The Benjamin–Hochberg method was used for *P* value corrections in the case of multiple testing. Further information on statistical tests and R packages utilized for the analyses are detailed in Appendices S1 and S2 (https://doi.org/10.6084/m9.figshare.13280435.v3).

## Results

### Clinical, epidemiological and microbiological features of participants

**Fig. 1.**
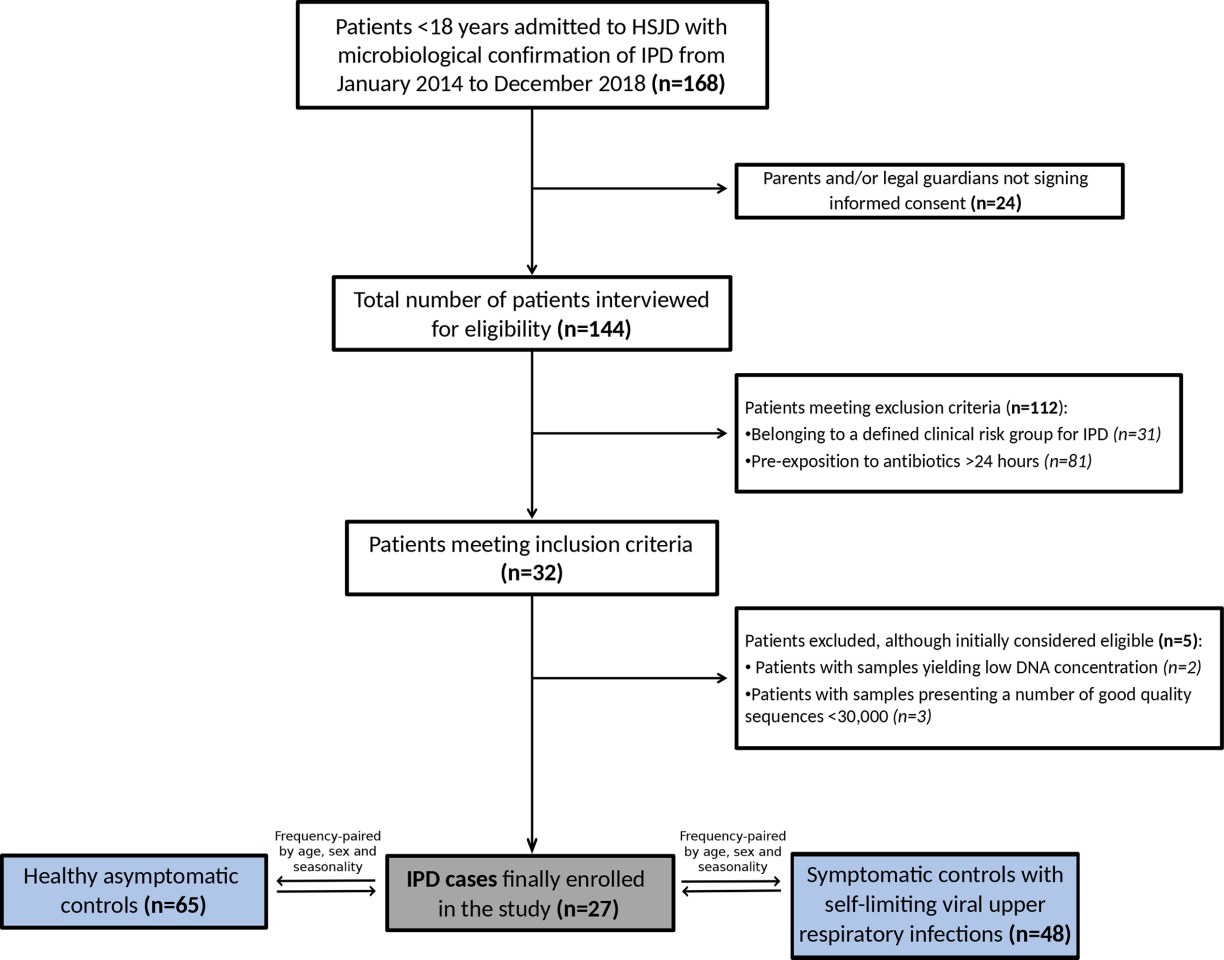
Flowchart of patients. Description of the IPD case selection process.

A total of 27 IPD cases, 48 viral URTI controls and 65 healthy/asymptomatic controls were finally enrolled in the study (Fig. 1). The median age of cases was 33 months (IQR: 19.0–49.5 months) and 55.6% were female. The main clinical IPD manifestation was bacteraemic pneumonia, which accounted for 85.2% (*n*=23) of cases, including complicated pneumonia with empyema (*n*=16, 59.3%) and non-complicated pneumonia (*n*=7, 25.9%). In addition, meningitis and occult bacteraemia occurred in 11.1% (*n*=3) and 3.7% (*n*=1) of cases, respectively. Detection of *S. pneumoniae* in normally sterile body fluids was performed by culture (*n*=3, 11.1%), PCR (*n*=16, 59.2%) or both culture and PCR (*n*=8, 29.6%). Invasive sample types positive for *S. pneumoniae* included blood (*n*=25, 92.6%), pleural fluid (*n*=10, 37.0%) and cerebrospinal fluid (*n*=3, 11.1%) specimens. The median length of hospital stay was 11 days (IQR: 8.0–15.0 days). A proportion of 14.8% (*n*=4) of cases required admission to the PICU, with a median stay of 2.5 days (IQR: 1.7–3.5 days). The majority of IPD cases evolved favourably (*n*=24, 88.9%), except for three patients who presented sequelae 6 months after episode onset (11.1%; two cases of meningitis and one case of complicated pneumonia). No deaths occurred in our study population.

The three groups of the study did not significantly differ in age, sex or seasonality (Table 1). However, IPD cases were more likely to present a history of gastrointestinal symptoms in the previous month, high leucocyte levels or low haemoglobin levels at admission blood testing than viral URTI and healthy/asymptomatic controls (Table 1). In addition, median duration of fever before NPA collection was higher in IPD cases (120.0 h, IQR 78.0–144.0 h) than in viral URTI controls (24.0 h, IQR 12.0–48.0 h; *P*<0.001). Kindergarten attendance and non-white ethnicity were significantly associated with viral URTI controls in comparison to those who were healthy/asymptomatic.

**Table 1.** Characteristics of the study groups

Parameter	IPD group (*n*=27)	URTI control group (*n*=48)	Healthy control group (*n*=65)	Global *P* value^a^	IPD – URTI^a^	IPD – healthy	URTI – healthy
**Demographic and epidemiological parameters**							
Median age, months (IQR)	33 (19.0–49.5)	24.5 (14.7–45.0)	31 (19.0–43.0)	0.85	–	–	–
Gender, female (%)	15/27 (55.6)	24/48 (50.0)	28/65 (43.1)	0.53	–	–	–
Mean birth weight, g (sd)^b^	3259.6 (506.9)	3108.7 (538.1)	3296.7 (521.2)	0.26	–	–	–
Median gestational age, weeks (IQR)^c^	40 (38.2–40.4)	39.3 (38.0–40.0)	40 (39.0–40.0)	0.32	–	–	–
Median house surface per inhabitant, m^2^ (IQR)^d^	20 (18.1–28.3)	20 (17.1–23.3)	22.5 (18.1–26.7)	0.38	–	–	–
Seasonality, samples collected during viral season (%)^i^	17/27 (63.0)	27/48 (56.3)	30/65 (46.1 %)	0.29	–	–	–
Ethnicity, white (%)^j^	16/24 (66.7)	24/48 (50.0)	55/64 (85.9)	<0.001***	0.27	0.16	<0.001***
Delivery mode, C-section (%)	6/23 (26.1)	15/48 (31.2)	20/62 (32.2)	0.86	–	–	–
Breastfeeding (%)	23/26 (88.5)	38/48 (79.2)	49/65 (75.4)	0.40	–	–	–
Median breastfeeding duration, months (IQR)^e^	6.5 (1.6–12.0)	6.0 (1.7–12.0)	6.0 (1.0–18.0)	0.98	–	–	–
Breastfeeding duration ≥6 months (%)	14/26 (53.8)	29/48 (60.4)	33/65 (50.8)	0.47	–	–	–
Kindergarten attendance (%)	15/26 (57.7)	31/47 (65.9)	19/65 (29.2)	0.002**	0.36	0.08(.)	<0.001***
Schooled (%)	21/26 (80.8)	36/47 (76.6)	43/65 (66.1)	0.26	–	–	–
Household members under 5 years (%)	9/25 (36.0)	20/46 (43.5)	15/60 (25.0)	0.13	–	–	–
Smoking habits in the household (%)	8/26 (30.8)	15/48 (31.2)	36/64 (56.2)	0.02*	1.00	0.08(.)	0.04*
Educational level, basic (%)	3/19 (15.8)	9/47 (19.1)	6/57 (10.5)	0.49	–	–	–
≥1 dose of PCV (%)	15/27 (55.5)	34/48 (70.8)	49/65 (75.4)	0.17	–	–	–
Gastroenteritis in the previous month (%)	9/25 (36.0)	5/48 (10.4)	6/62 (9.7)	0.003**	0.04*	0.03*	1.00
**Analytical and blood parameters**							
Blood test – median haemoglobin, g dl^−1^ (IQR)^f^	10.7 (10.1–11.7)	12.1 (11.7–12.6)	12.5 (11.9–13.1)	<0.001***	0.09(.)	<0.001***	0.41
Blood test – median leucocytes, thousandmm^−3^ (IQR)	16.5 (10.7–19.8)	5.7 (5.1–8.6)	9.0 (6.4–10.0)	<0.001***	<0.01**	<0.001***	0.17
**Microbiological parameters**							
NP pneumococcal carriage (%)	27/27 (100)	25/48 (52.1)	40/64 (62.5)	<0.001***	<0.001***^h^	<0.01**^h^	0.36^h^
Median NP pneumococcal load, log_10_ copies ml^−1^ (IQR)^g^	6.33 (5.3–6.7)	3.53 (0–6.1)	4.45 (0–6.1)	<0.001***	<0.001***	<0.001***	<0.001***
NP pneumococcal serotype covered by PCV13 vaccination (%)	14/27 (51.8)	4/25 (16.0)	5/40 (12.5)	0.001***^h^	0.02*^h^	<0.01**^h^	0.73^h^
NP pneumococcal serotype with high invasive disease potential (%)	14/27 (51.8)	0/25 (0.0)	4/40 (10.0)	<0.001***^h^	<0.001***^h^	<0.001***^h^	0.15^h^
DNA/RNA viral detection by multiplex PCR (%)^k^	22/27 (81.5)	48/48 (100)	39/65 (60.0)	–	–	0.05*^h^	–
DNA/RNA viral detection >2 viruses by multiplex PCR (%)^k^	9/27 (33.3)	28/48 (58.3)	10/65 (15.4)	–	–	0.10(.)	–
Human rhinovirus/enterovirus (%)^k^	16/27 (59.2)	27/48 (56.2)	27/65 (41.5)	–	–	0.19	–
Human adenovirus (%)^k^	3/27 (11.1)	15/48 (31.2)	7/65 (10.8)	–	–	1.00^h^	–
Human bocavirus (%)^k^	4/25 (16.0)	12/48 (25.0)	3/65 (4.6)	–	–	0.09(.)^h^	–
Human coronaviruses NL63, OC43, 229E (%)^k^	2/27 (7.4)	6/48 (12.5)	5/6 5(7.7)	–	–	1.00^h^	–
Human influenza A virus (%)^k^	1/26 (3.8)	3/48 (6.2)	0/65 (0.0)	–	–	0.29^h^	–
Human influenza B virus (%)^k^	2/26 (7.7)	3/48 (6.2)	2/65 (3.1)	–	–	0.32^h^	–
Human influenza A and B virus (%)^k^	3/26 (11.5)	6/48 (12.5)	2/65 (3.1)	–	–	0.14^h^	–
Human parainfluenza 1 virus (%)^k^	0/27 (0.0)	4/48 (8.3)	0/65 (0.0)	–	–	–	–
Human parainfluenza 3 virus (%)^k^	1/27 (3.7)	5/48 (10.4)	1/65 (1.5)	–	–	0.50^h^	–
Human parainfluenza 4 virus (%)^k^	0/27 (0.0)	0/48 (0.0)	3/65 (4.6)	–	–	0.55^h^	–
Human parainfluenza 1,3,4 viruses (%)^k^	1/27 (3.7)	9/48 (18.7)	4/65 (6.1)	–	–	1.00	–
Human respiratory syncytial virus A and B (%)^k^	4/26 (15.4)	4/48 (8.3)	2/65 (5.1)	–	–	0.05*^h^	–
Human metapneumovirus (%)^k^	1/27 (3.7)	6/48 (12.5)	1/65 (1.5)	–	–	0.50^h^	–

^a^ANOVA and Kruskal–Wallis tests were used for parametric and non-parametric continuous variables, respectively. Chi-square test was used for categorical variables. Specific group differences among quantitative variables were pointed out by *post hoc* Tukey HSD or pairwise Wilcoxon tests with Benjamin–Hochberg corrections for multiple testing in case of parametric and non-parametric variables, respectively. Pairwise Chi-squared tests were used for categorical variables.

^b^Comparisons performed on 25 IPD cases, 47 URTI controls and 65 healthy controls.

^c^Comparisons performed on 26 IPD cases, 47 URTI controls and 65 healthy controls.

^d^Comparisons performed on 23 IPD cases, 44 URTI controls and 62 healthy controls.

^e^Compaisons performed on 26 IPD cases, 48 URTI controls and 62 healthy controls.

^f^Comparisons performed on 25 IPD cases, 6 URTI controls and 41 healthy controls.

^g^Comparisons performed on 27 IPD cases, 48 URTI controls and 64 healthy controls. Subjects with negative PCR were assumed to present 0 pneumococcal genome copies/ml^−1^. A sum of a small pseudocount (value +1) was applied to this variable prior to log_10_ transformation.

^h^Fisher exact test and pairwise Fisher exact test were performed for categorical variables instead of Chi-square tests in case of ≥25% of cells presented expected frequencies ≤5.

iViral season was defined as the period of time corresponding to influenza A and RSV circulation over the basal levels according to the Surveillance Plan of ARIs in Catalonia (PIDIRAC) (https://canalsalut.gencat.cat/ca/professionals/vigilancia-epidemiologica/pla-dinformacio-de-les-infeccions-respiratories-agudes-a-catalunya-pidirac/) and reports from the Hospital Surveillance Network for RSV in Catalonia (Vall d’Hebrón Hospital) (https://hospital.vallhebron.com/ca/actualitat/publicacions/informe-xarxa-de-vigilancia-hospitalaria-de-vrs).

^j^The white group included individuals of European origin, while non-white referred to African, African American, Asian, Latin American and mixed ethnic groups.

^k^ *P* values reporting significance for comparisons between healthy and IPD groups. URTI control group was not considered.

Significance values: ***≤0.001, **≤0.01, *≤0.05, (.)≤0.1 (trend).

NP, Nasopharyngeal; RSV, Respiratory Syncytial Virus.

Pneumococcal colonization was strongly related to the IPD group: 100.0 % (*n*=27) of cases were pneumococcal nasopharyngeal carriers compared to 52.1 % (*n*=25) of viral URTI controls and 62.5 % (*n*=40) of healthy/asymptomatic controls. Higher pneumococcal nasopharyngeal load values were also quantified in the IPD group compared to the two control groups. Furthermore, pneumococcal serotypes detected among children with IPD were significantly more likely to be included in PCV13 and to present higher invasive disease potential than serotypes detected in control groups. Notably, none of the serotypes identified among viral URTI controls were classified as high invasive disease potential serotypes. A higher prevalence of viral infection was associated with IPD cases compared to healthy/asymptomatic controls. Table 1 details the clinical, epidemiological and microbiological characteristics of participants.

### Nasopharyngeal microbiota parameters according to respiratory health status and environmental factors

A total of 11 769 440 good quality sequences were obtained from samples and negative controls (Table S1). Overall, these sequences were clustered into 726 OTUs; however, 124 OTUs were considered as contaminant OTUs and removed. Such contaminant OTUs accounted for 10 375 sequences in negative controls (median=419, IQR=149–711) and 57 847 sequences in samples (median=57, IQR=16–175), representing a median of 57.1 % of the sequences found in negative controls (IQR=40.80–67.50) and a median of 0.07% of the sequences found in samples (IQR=0.02–0.27). There were only two samples with a higher proportion of contaminant sequences: 33% of sequences were assigned to contaminant *Phyllobacterium* (OUT 24) in one sample, while 13 % of sequences were assigned to contaminant *Pseudomonas* (OTU 66) in another sample (Fig. S1).

Finally, 602 OTUs accounting for 11 696 096 sequences among samples included in the study (median=73 553, IQR: 55 974–95 915) were considered for downstream analyses. A summary of the number of OTUs and sequences obtained at each clean-up step can be found in Table S1.

**Fig. 2.**
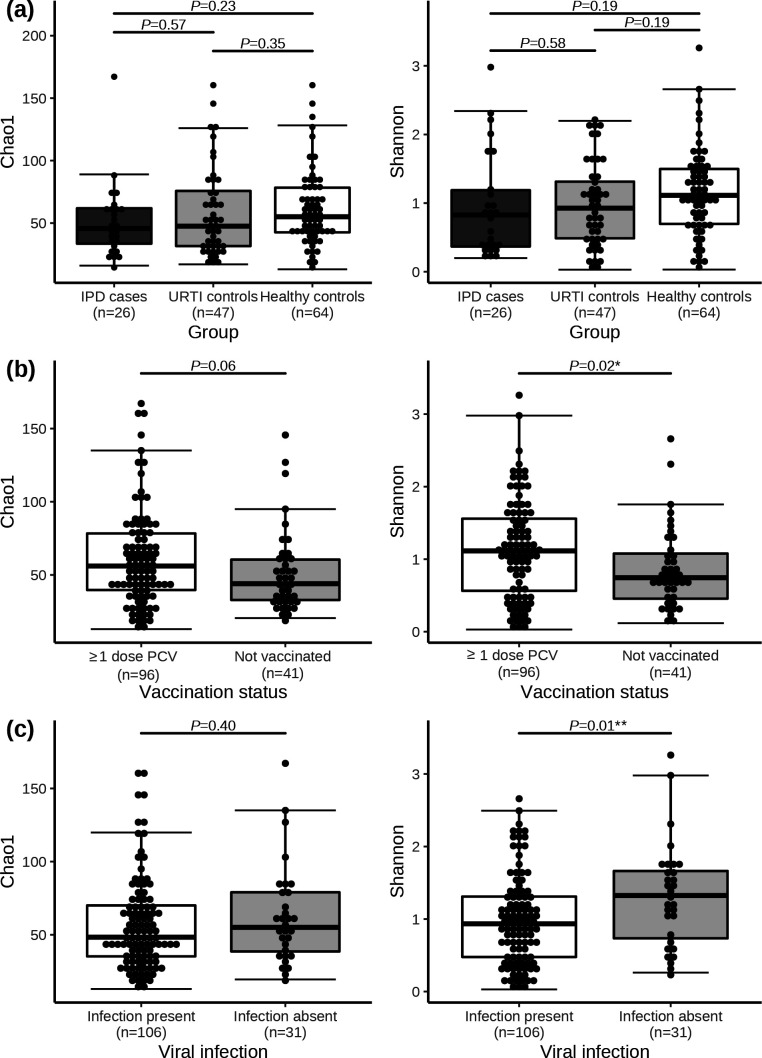
Comparison of Chao1 richness and Shannon diversity indexes. Boxplots with median and IQR are used for representing Chao1 richness and Shannon diversity values of nasopharyngeal samples according to respiratory health status (a), vaccination status (b) and viral infection (c). *P* values of significance asterix-tagged depending on strength of significance: ***P≤0.001, **P≤0.01 and *P≤0.05.

Alpha-diversity analysis enabled the characterization of groups in terms of bacterial species richness and diversity. IPD cases (Chao1 median=45.7, IQR=33.6–61.9/Shannon median=0.83, IQR=0.37–1.19) and URTI controls (median Chao1=47.5, IQR=31.6–75.7/Shannon median=0.92, IQR=0.49–1.31) presented lower richness and diversity values in comparison to healthy/asymptomatic controls (Chao1 median=55.1, IQR=42.6–78.4/median Shannon=1.11, IQR=0.70–1.50). Despite the trends being observed, such differences did not achieve statistical significance (Fig. 2a). Besides the groups of study, children with ≥1 dose of PCV were associated with a more diverse microbiota as measured by Shannon index (median=1.11, IQR=0.56–1.56) when compared to non-vaccinated children (median=0.74, IQR=0.46–1.08) (Fig. 2b). However, a complex interaction existed between vaccination status and study groups [permutational analysis of covariance (ANCOVA); *P*=0.03]; such differences of diversity according to vaccination status were mainly observed among IPD and URTI control groups. Virally infected children were associated with lower Shannon diversity values (median=0.93, IQR=0.48–1.31) in comparison to those non-infected (median=1.32, IQR=0.73–1.66) (Fig. 2c). Age, gender, breastfeeding duration according to WHO recommendations and sequencing run were non-determinant variables for bacterial Chao1 richness or Shannon diversity (Fig. S2).

Beta-diversity analyses revealed differences in nasopharyngeal microbiota composition of children according to respiratory health status using a Bray–Curtis (PERMANOVA R^2^=3.6%, *P*=0.01; PERMDISP *P*=0.63) dissimilarity matrix (Fig. S3). *Post hoc* comparisons showed that IPD cases and healthy controls presented the most dissimilar microbiota profiles (R^2^=3.8%, *P*=0.04). A trend for significance in profile composition was observed for healthy and viral URTI controls (R^2^=2.0%, *P*=0.08), as well as for IPD cases and viral URTI controls (R^2^=2.5%, *P*=0.11). Additionally, microbiota composition was influenced by breastfeeding ≥6 months (R^2^=2.2%, *P*=0.02), vaccination status (R^2^=2.7%, *P*=0.007), viral infection (R^2^=3.4 %, *P*=0.003) and nasopharyngeal pneumococcal load (R^2^=3.7 %, *P*=0.002) (Fig. S3). Neither age nor gender nor sequencing run were related to relevant modifications of beta-diversity (Fig. S3). Further analyses with Jaccard and weighted Unifrac measures corroborated these results (Figs S4 and S5), and went even further by demonstrating deeper differences on microbiota composition between IPD and URTI groups when using a phylogenetic-based distance as the latter (R^2^=4.5 %, *P*=0.03; Fig. S5).

**Fig. 3.**
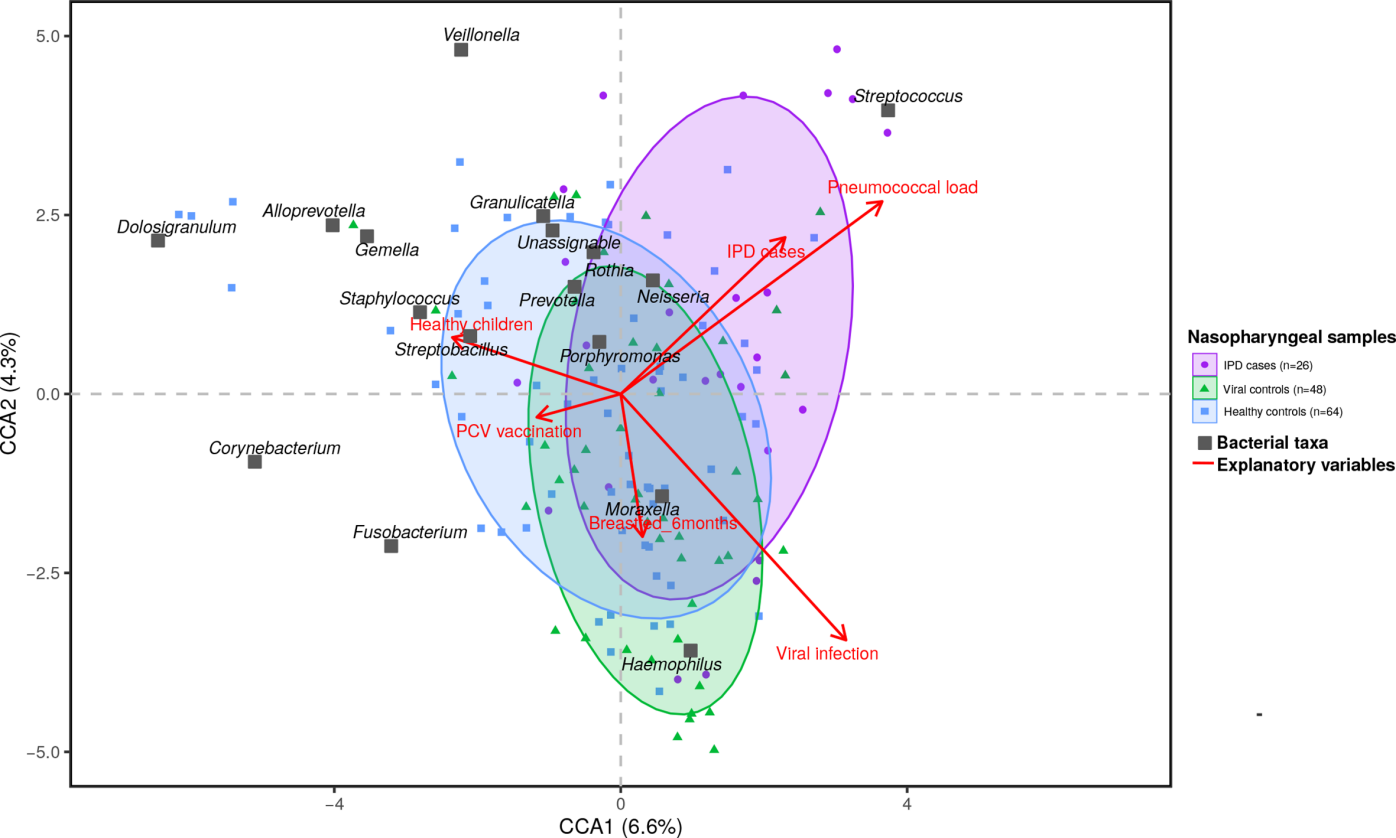
Triplot of CCA showing distribution of nasopharyngeal samples with reference to bacterial genera and explanatory variables. Nasopharyngeal samples are represented by purple dots, green triangles and blue squares corresponding to nasopharyngeal samples from IPD children, children with self-limiting viral infections and healthy children, respectively. For clarity, ellipses are drawn containing 75% of nasopharyngeal samples from each study group and coloured accordingly. The red arrows indicate the direction and strength (length) of the explanatory variables. The dark grey squares correspond to the peaks of higher abundance of each bacterial genus. Genera labels are written next to the squares.

CCA assessed the relevance and relation of these variables influencing beta-diversity with the nasopharyngeal microbiota composition (Fig. 3). The total inertia of the CCA model was 1.33, of which 0.16 corresponded to constrained inertia. The CCA corroborated that a proportion of the constrained variance observed in the bacterial communities was driven by the significant variables influencing beta-diversity (CCA model, *P*=0.001). The axis CCA1 (*P*=0.001), CCA2 (*P*=0.007) and CCA3 (*P*=0.04) were the main axes capturing a significant proportion of constrained variation (14.1%). The results indicated that IPD cases (*P*=0.01), higher pneumococcal loads (*P*=0.001) and viral infections (*P*=0.001) were the most important factors shaping the structure of microbial communities in this study, as denoted by the exhibition of the longest arrows. It can be observed that *Streptococcus* falls close to the vector of IPD group and pneumococcal load, indicating potential strong positive associations. However, *Haemophilus* was associated with the vector of viral infections (Fig. 3). Inclusion in the healthy/asymptomatic control group (*P*=0.02) or PCV13 vaccination (*P*=0.005) were pointing to a cluster of taxa represented by *Dolosigranulum *, *Corynebacterium* and a series of oral bacteria. Also to be noted, *Moraxella* was relatively close to breastfeeding ≥6 months (*P*=0.02) (Fig. 3).

### Identification of bacterial biomarkers associated with respiratory health status and environmental factors

The most abundant genera were *Moraxella *, *Haemophilus *, *Streptococcus *, *Dolosigranulum *, *Corynebacterium *, *Staphylococcus *, *Veillonella* and *Neisseria *, with median relative abundances of 24.60, 15.00, 8.50, 0.26, 0.06, 0.02, 0.02 and 0.02%, respectively. However, their distribution varied according to respiratory health status: *Streptococcus* (26.30%), *Haemophilus* (22.82%) and *Moraxella* (27.58%) were the most abundant genera in IPD cases, viral URTI controls and healthy/asymptomatic controls, respectively. At a deeper taxonomic level, the most abundant taxa in each group corresponded to *S. pneumoniae* (OTU 1; 19.99%), *H. influenzae* (OTU 5; 19.61%) and *Moraxella* (OTU 3; 21.02%), respectively. All OTUs and genera detected by group, together with their median relative abundances, are shown in Tables S2 and S3.

**Fig. 4.**
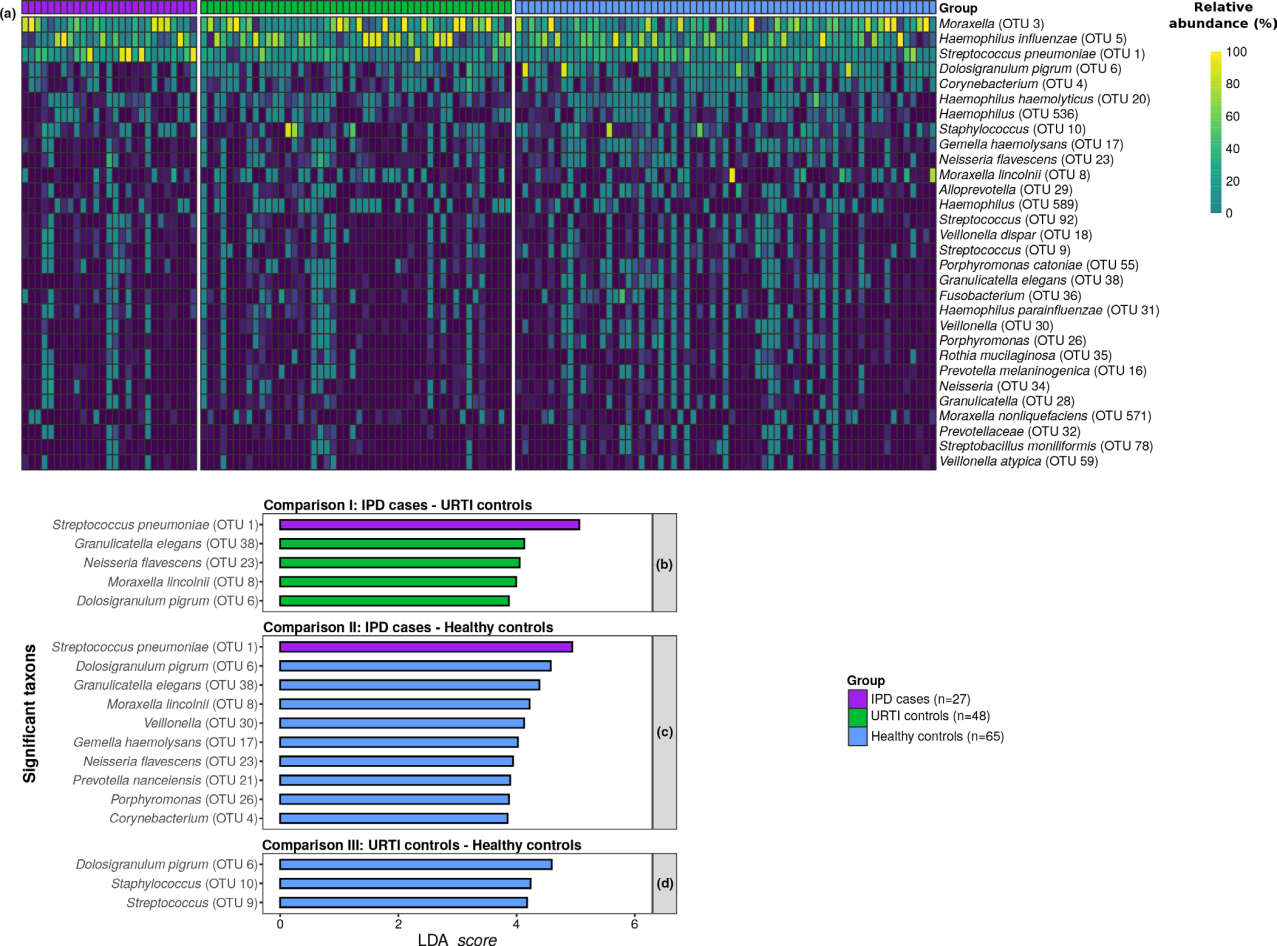
Relative abundance of OTUs according to respiratory health status. (a) Relative abundance heatmap of specific OTUs across samples. Samples belonging to the same group were put together in the *x* axis. OTUs were ordered according to their median relative abundance values. (b–d) In addition, LEfSe identified bacterial OTUs with statistically significant differences in their relative abundance between IPD cases and viral URTI controls (b), IPD cases and healthy controls (c), and between both control groups (d).

Differential distribution analyses with LEfSe identified *S. pneumoniae* (OTU 1) as the main taxon overrepresented in the nasopharyngeal microbiota of children with IPD (Fig. 4a–c). However, control groups presented a significantly higher abundance of different bacteria. *Dolosigranulum pigrum* (OTU 6) (Fig. 4b, c) was associated with controls, and healthy children exhibited the highest abundance levels of this bacterium (Fig. 4c, d). *Moraxella lincolnii* (OTU 8), *Neisseria flavescens* (OTU 23) and *Granulicatella elegans* (OTU 38) were also associated with both control groups (Fig. 4b, c). In turn, the nasopharyngeal microbiota of healthy/asymptomatic children was enriched in *Corynebacterium* (OTU 4) and several taxa commonly found in the oral niche, such as *Veillonella* (OTU 30), *Gemella haemolysans* (OTU 17) and *Prevotella nanceiensis* (OTU 21), when compared to IPD (Fig. 4c). Consistent results were found at the genus level (Fig. S6).

LEfSe also corroborated the association of bacterial genera from the oral cavity, such as *Alloprevotella *, *Porphyromonas *, *Veillonella *, *Gemella* and *Granulicatella *, with children who received at least one dose of PCV. *Haemophilus* was overrepresented in the group of children with viral infections, while *Dolosigranulum *, *Corynebacterium* or *Gemella* were significantly less abundant in this group. No specific bacterial genus was associated with maternal breastfeeding duration ≥6 months (Fig. S7).

### Bacterial correlations

**Fig. 5.**
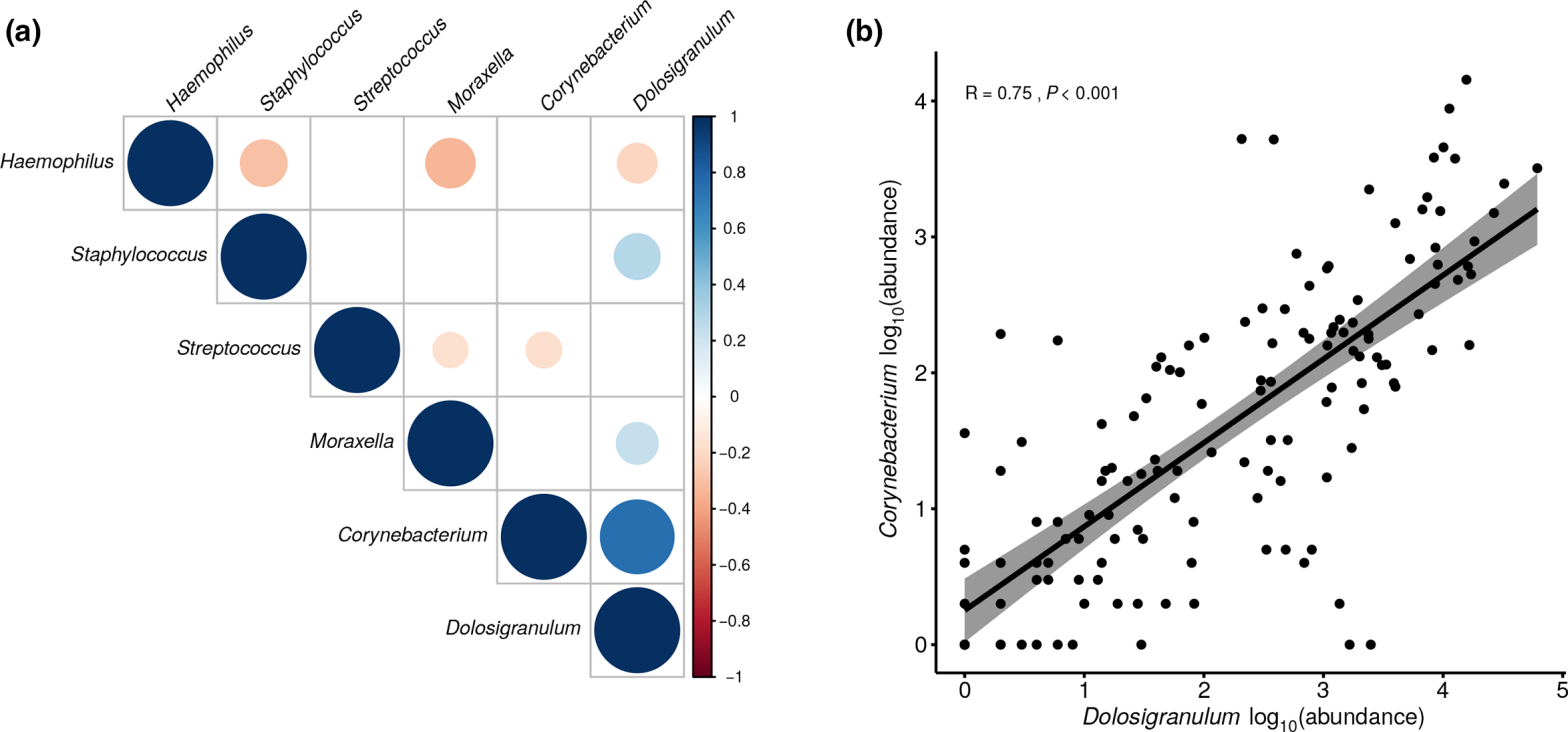
Bacterial genera correlations. A Spearman’s correlation matrix between dominant genera detected in nasopharyngeal samples is shown in (a). Only significant correlations are shown (*P*<0.05). (b) A scatter plot of the Spearman’s correlation between two specific genera: *Dolosigranulum* and *Corynebacterium *.

Spearman’s correlations revealed significant interrelations between the dominant bacteria of the nasopharyngeal microbiota (Fig. 5a). *Streptococcus* was found to be negatively associated with *Corynebacterium* and *Moraxella *. The latter correlation was mainly due to negative associations between *M. lincolnii* (OTU 8) and *S. pneumoniae* (OTU 1) (Table S4). The genus *Haemophilus* was inversely correlated to the presence of *Dolosigranulum *, *Moraxella* and *Staphylococcus *. Positive correlations were detected as well between *Dolosigranulum* and *Moraxella *. However, the strongest correlation corresponded to that of *Dolosigranulum* with *Corynebacterium *, displaying a high lineal relation with coefficient of 0.75 % (*P*<0.001) (Fig. 5b). Significant and non-significant bacterial interrelations are shown in Tables S4 and S5 at OTU and genus level.

### Case–control classification using RF classifier

**Fig. 6.**
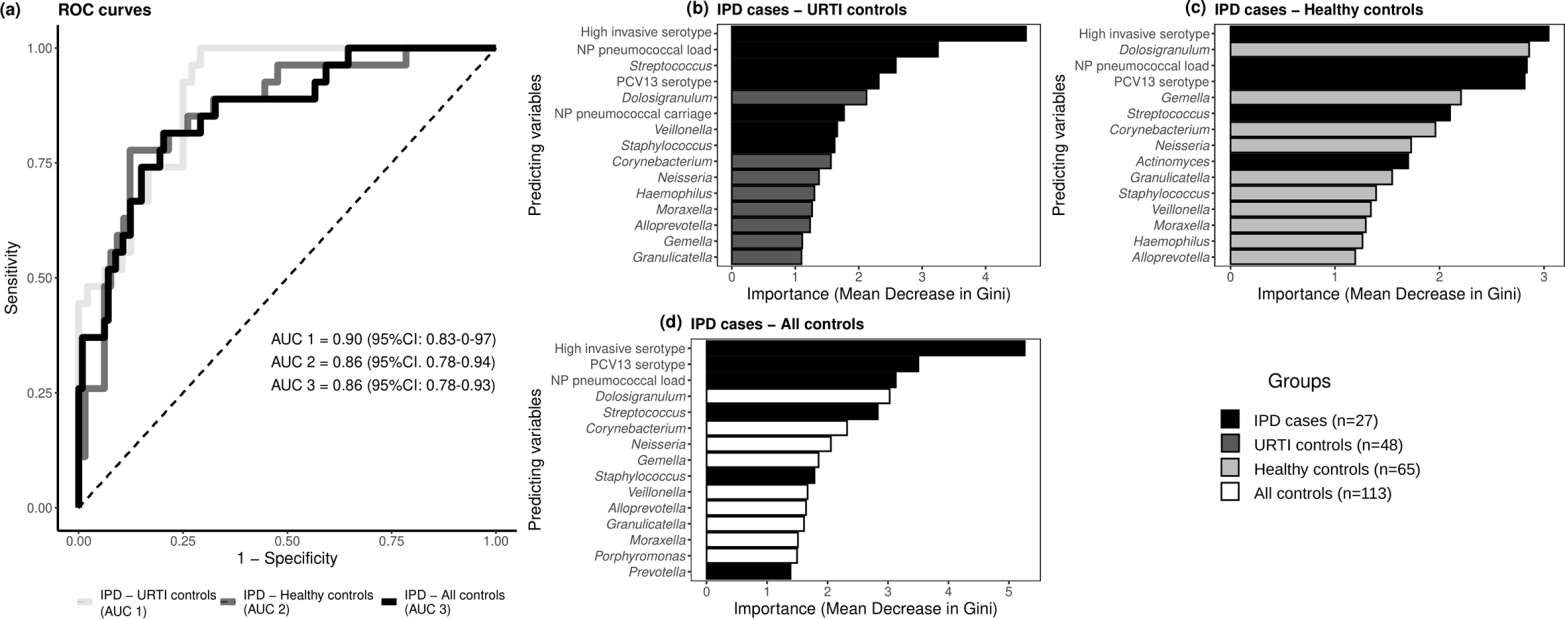
Performance of RF classifier models. RF models were utilized for distinguishing (**i**) IPD cases from URTI controls, (ii) IPD cases from healthy controls, and (iii) IPD cases from all controls using pneumococcal and viral parameters as well as microbiota abundance values of OTUs. Comparison of the ROC curves of the different models in shown in (a). In addition, the contribution of the variables to the performance of the three models is shown in (b, c, d), through the representation of the mean decrease in Gini index of each variable.

RF models showed good performance in discriminating cases from controls by combining only microbiological parameters assessed on nasopharyngeal samples. Receiver operating characteristic (ROC) curves of the models showed AUCs of 0.90 [95% confidence interval (CI): 0.83–0.97], 0.86 (95% CI: 0.78–0.94) and 0.86 (95% CI: 0.78–0.93) for model I (IPD cases versus viral URTI controls), model II (IPD cases versus healthy/asymptomatic controls) and model III (IPD cases versus all controls), respectively (Fig. 6a). Important predictors related to IPD were pneumococcal colonization (essentially colonization by serotypes with high invasive disease potential and/or serotypes covered by PCV13 vaccination), high nasopharyngeal pneumococcal load quantified by quantitative real-time PCR and high abundance of the genus *Streptococcus* as determined by 16S rRNA gene sequencing (Fig. 6b, c, d). In turn, the most important predictor associated with controls was a high abundance of *Dolosigranulum *, followed by a high abundance of other genera such as *Corynebacterium *, *Neisseria *, *Moraxella *, *Gemella* and others (Fig. 6d). Nasopharyngeal pneumococcal colonization (independently of the serotype detected) was considered important for classification in model I (IPD vs viral URTI) (Fig. 6b). Very similar results were obtained at the OTU level (Fig. S8).

## Discussion

This study unveiled significant differences in nasopharyngeal microbiota structure according to respiratory health status, and identified a number of bacterial species potentially protective against IPD. Alpha-diversity analyses showed the highest species richness and diversity levels associated with the healthy control group, despite no statistical significance being obtained. There is a widely accepted idea that loss of microbial diversity is associated with human disease, while a healthy resilient microbiota relies on high richness and biodiversity [9]⁠. However, the present results may contradict in part the results of our previous study showing a highly diverse nasopharyngeal microbiota associated with IPD [25]. ⁠Differences between diversity patterns linked to IPD or health in our two studies may be explained by distinct study designs; while the previous study included patients exposed to β-lactam antibiotic treatment during a mean period of 4 days, this current study was exclusively performed with patients exposed ≤24 h to antibiotics. A longitudinal study performed on children with cystic fibrosis supports our hypothesis by demonstrating a transient increase of diversity at 3 days of β-lactam antibiotic therapy that normalizes at day 8–10 [48].

A healthy microbiota profile was further characterized as mainly dominated by *Moraxella* and overrepresentation of *D. pigrum *, *M. lincolnii *, *Corynebacterium* and a great variety of bacterial species typically found in the oral cavity [49]. This is in line with a previous study by our group that found a lower prevalence of IPD cases among the nasopharyngeal-type dominated by *D. pigrum. D. pigrum* belongs to the group of lactic acid bacteria (LAB), which produce lactate as the main metabolite of glucose [50]. LAB play numerous beneficial functions in the gut; inhibition of overgrowth of pathogenic micro-organisms, degradation of mycotoxins or stimulation of the immune system, among others [51]. The results from the present study confirm that *D. pigrum* is enriched in the nasopharynx of healthy children, suggesting that *D. pigrum* could carry out similar functions in the respiratory microbiome, in combination with other beneficial bacteria like *Corynebacterium *. In this regard, we noted a highly positive correlation between the two bacterial species as well as negative associations with main bacterial pathobionts. This finding may indicate that both species could act together to contain pathogenic outgrowth and is aligned with results from Brugger *et al.*, reporting that inhibition of *S. pneumoniae* by *D. pigrum* requires the presence of *Corynebacterium* [52]. Other studies have also documented the presence of both *Dolosigranulum* and *Corynebacterium* in healthy phenotypes [8, 17, 53]. Such a close relationship may be explained in part by the acidification of the surrounding environment with lactate produced by *D. pigrum *, which is favourable for *Corynebacterium* [5]. The higher frequency of *Veillonella* in healthy children compared to IPD cases also supports the existence of a lactate-rich environment, given that this bacterium utilizes lactate as a carbon source [54]. The role of *Moraxella* in respiratory health is more controversial: although several studies consistently report that *Moraxella* enrichment is linked to increased susceptibility and severity in ARIs [14, 23, 24, 55], others describe high *Moraxella* abundances associated with health and less severe manifestations of respiratory infections [10, 22, 56]. These conflicting results may derive from the distinct *Moraxella* species detected across studies, most of them not achieving species level taxonomic resolution. In fact, it has been described that by the age of 6 months, two distinct *Moraxella *-dominated microbiota profiles exist: one highly suggestive of *Moraxella catarrhalis* and the other corresponding to a *M. lincolnii* species [10]. While the former has been typically related to ARI episodes, the latter is a typical commensal bacterium from the URT whose relationship with respiratory health is not clear [11, 55]. To our knowledge, there is only one study reporting *M. lincolnii* as a protective bacterium against recurrent acute otitis media [17] and our results corroborate its association to healthy phenotypes.

The origin of potentially beneficial bacteria remains uncertain, some studies pointing to human milk [12, 57]. In this respect, we found that a longer duration of maternal breastfeeding was associated with significant changes of microbiota structure, but we were not able to demonstrate a significant enrichment of potentially beneficial bacteria in children breastfed for at least 6 months. Instead, we found decreased abundances of several anaerobic bacteria, including *Alloprevotella *, *Veillonella* and *Porphyromonas *, similarly to that which occurred in children breastfed for 6 weeks [12] or breastfed for 3 months [58]. Although they are generally considered as commensal bacteria of the oral cavity, these bacteria in conjunction with other bacterial pathogens, such as *S. pneumoniae *, *H. influenzae* or *Mycoplasma pneumoniae *, may play an important role in respiratory infections [59]. In addition to maternal breastfeeding, vaccination strategies have also been related to beneficial effects through the modulation of commensal microbiota from the URT. In our study, pneumococcal vaccination with ≥1 dose was significantly associated with a highly diverse microbiota structure, including micro-organisms typically found as oral commensals. It is plausible that the empty nasopharyngeal niche left by *S. pneumoniae* removal may be occupied by micro-organisms from the surrounding environment, including the oral cavity [46]. The blp-like streptococcal bacteriocins, for example, are known to be active against many oral organisms [60], and many nutritional and antagonistic interactions have been found between streptococci and other oral bacteria [61]. Although exploratory analyses have been performed in this study, future research specifically designed to study the effect of vaccination on nasopharyngeal microbiota composition should further explore this topic, given the complex relations and interactions established between vaccination and respiratory health status.

We characterized a nasopharyngeal microbiota profile dominated by *Streptococcus* and displaying a low number of taxa that was associated with IPD. Differential abundance testing and specific pneumococcal PCR confirmed enrichment of *S. pneumoniae *, highlighting a direct expectable relation of this pathobiont with the disease. However, the role of *S. pneumoniae* transcends a specific infection, and has been associated with increased susceptibility and severity to multiple respiratory diseases of different aetiology [22, 23, 62–65]. In this study, such enrichment coexisted with the underrepresentation of potential beneficial bacteria, an event that has been observed in longitudinal studies before and during ARIs [66], and an increased rate of viral infection among IPD cases in comparison to healthy children. Direct viral–bacterial interactions implicated in the pathogenesis of respiratory infections have already been described [5]. The mechanism by which viruses enhance the occurrence of serious bacterial infections and potentially drive symptoms’ severity is still far from being understood. It is believed that viral species reduce ecological biodiversity, promoting an imbalanced state of the nasopharyngeal microbiota, which facilitates pathogenic bacteria invasion [67]. However, others postulate that loss of microbial biodiversity is by itself a predisposing factor for viral acquisition [67]. Of particular note, we found that viral infection is an important factor that modulates bacterial diversity, but does not determine serious bacterial infection by itself. In this regard, the viral URTI control group was not associated with clinically relevant phenotypes, despite exhibiting an *Haemophilus *-dominated microbiota commonly related to increased susceptibility to and severity of viral ARIs [14, 23, 62, 63]. We speculate that the self-limiting course of viral infections observed in the viral URTI group may be explained by the maintenance of a relatively balanced microbiota with intermediate microbial composition between healthy and IPD groups, as demonstrated by beta-diversity analyses. This specific microbiota composition could counteract the triggering effect of concurring pathogenic bacteria and viruses maintaining a localized infection. Our hypothesis is also supported by the presence of potential beneficial commensals such as *D. pigrum* and *M. lincolnii* in the microbiota compositions characterized among controls with viral URTI. Altogether, these data suggest that a complete dysbiosis has not yet been produced in this specific group. Retaining some beneficial species could be key for preserving certain ecological homeostasis when microbiota disturbances are produced. Further evidence is provided by other studies that have reported gradual changes in nasopharyngeal microbiota composition according to disease severity [53, 62].

Integration of nasopharyngeal microbiological parameters into multivariate classification models, including microbiota species abundance, enabled accurate differential diagnosis of IPD cases, whose main clinical manifestation was pneumonia in up to 85% of cases. This finding is especially relevant, since the WHO defines pneumonia and subsequent treatments solely based on clinical signs and symptoms because the aetiological diagnosis is only achieved in a low number of pneumonia cases [68, 69]. Moreover, aetiological studies of pneumococcal pneumonia or pneumonia frequently caused by other bacterial pathobionts in children are especially challenging due to the difficulty in obtaining adequate sputum specimens for differentiating colonization from infection. For this reason, microbiological confirmation of infection relies on the collection of invasive samples causing patient inconvenience and reluctance [70]. Our results suggest the potential of using URT samples for pneumococcal detection and quantification, which could contribute to differential diagnosis of IPD even in populations with high colonization rates. Higher pneumococcal loads and pneumococcal carriage rates of 100% were found in IPD cases, supporting the idea that colonization and overgrowth may be prior steps before invasive disease occurrence [6]. Similarly, diverse evidence has unveiled the contribution of ecosystems dysbiosis to the pathogenesis of respiratory infections and respiratory diseases with a chronic inflammatory character in children, such as asthma, bronchiolitis or cystic fibrosis. In such dysbiotic states, pathogenic bacteria are enriched, while other beneficial bacteria are underrepresented [5, 64]. Our findings point to the same direction, not only pneumococcal parameters related to pathogenic outgrowth were predictors of disease but also the lower abundance of commensal bacteria. Of note, discrimination between IPD cases and viral URTI controls on the basis of nasopharyngeal microbiota characteristics is in agreement with previous studies that have highlighted the potential of microbiota analyses for clinical diagnosis of infectious diseases [71, 72]. The first 72 h from episode onset are especially challenging for the differential diagnosis of potentially life-threatening bacterial infections from banal viral infections due to the overlap in the presentation of symptoms. Moreover, in recent years, technological advances have allowed integration of 16S rRNA gene sequencing analysis into small, portable, fast, low-cost and easy-to-use sequencing devices [73]. In this context, microbiota-based diagnostics hold promise for early aetiological diagnosis and specific antimicrobial treatment of ARIs at the point of need; thus, positively effecting mortality and antimicrobial resistance reduction [74, 75].

This study presents several limitations. As a consequence of the case–control design that we adopted, adequate for an exploratory study, only associations could be drawn from outcomes, without establishing causality. In addition, a small sample size was analysed due to a strict selection of cases exposed ≤24 h to antibiotics, which may have reduced the statistical power for detection of smaller but real effects of respiratory health status on alpha-diversity measures. In contrast, we consider that this stringent selection criterion has enabled better characterization of microbiota compositions without the bias of antibiotic effect. However, the consideration of a single model of severe disease may limit the applicability of the results. Nevertheless, a recent study points out that developing bacterial infections have a common pathway, suggesting that the mechanism is the same independently of the clinical phenotype [22]. Lastly, species-level resolution by using short-read sequencing technologies remains a challenge for microbiota studies, being especially problematic for those sequences assigned to *Streptococcus* [76]. To minimize this limitation, we used accurate programs specifically designed for species-classification of 16S rRNA gene amplicons together with high confidence thresholds, as described in methodology [77]. Moreover, we performed a specific PCR targeting the *lytA* gene for detecting *S. pneumoniae* on nasopharyngeal samples, leading to the same results.

In conclusion, our results add new evidence of the close relation between the nasopharyngeal microbiota and human respiratory health. Potentially protective species were enriched in controls, suggesting their implication in preventing LRTIs and systemic infections. In addition, initial evidence of the potential of microbiota-based diagnostics for differential diagnosis of severe ARIs using non-invasive samples is reported.

## Supplementary Data

Supplementary material 1
