# Supplementary material for: Differential nasopharyngeal microbiota composition in children according to respiratory health status
Source: Microb Genom. 2021 Oct 26;7(10):000661. doi: 10.1099/mgen.0.000661 (PMC8627214; doi:10.1099/mgen.0.000661)
Supplement: Supplementary material 1 [file mgen-7-0661-s001.pdf]

SUPPLEMENTARY MATERIAL

Figure S1. Relative abundance of contaminant OTUs among negative controls and samples included in the study. In the figure, contaminant OTUs with mean relative abundance over 0.5% among negative controls are colored according to their species assignment (these OTUs account for the 87% and 74% of all contaminant sequences found in negative controls and samples, respectively). On the other hand, contaminant OTUs with mean relative abundance under 0.5% among negative controls are grouped under the category "*Other contaminants*". In grey color are depicted those OTUs not considered as contaminant.

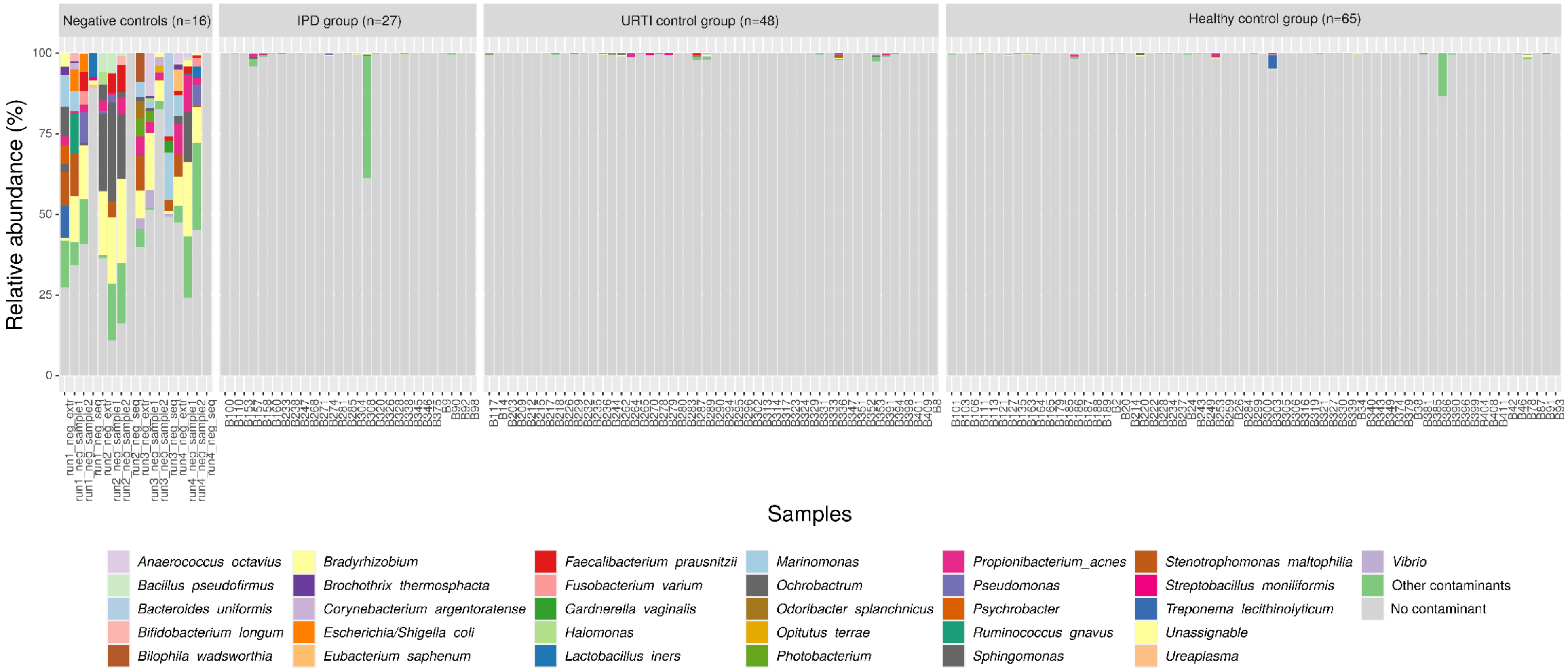

Figure S2. Alpha-diversity indexes according to different environmental variables. Alpha-diversity analyses according to age (S2-A), gender (S2-B), breastfeeding fulfillment of WHO recommendations (S2-C), and sequencing run (S2-D). A scatter plot of Spearman's correlation is utilized for visualizing the relation of Chao1 richness and Shannon diversity indexes with the quantitative variable age. Boxplots with median and IQR are used instead for representing diversity indexes according to the remaining categorical variables.

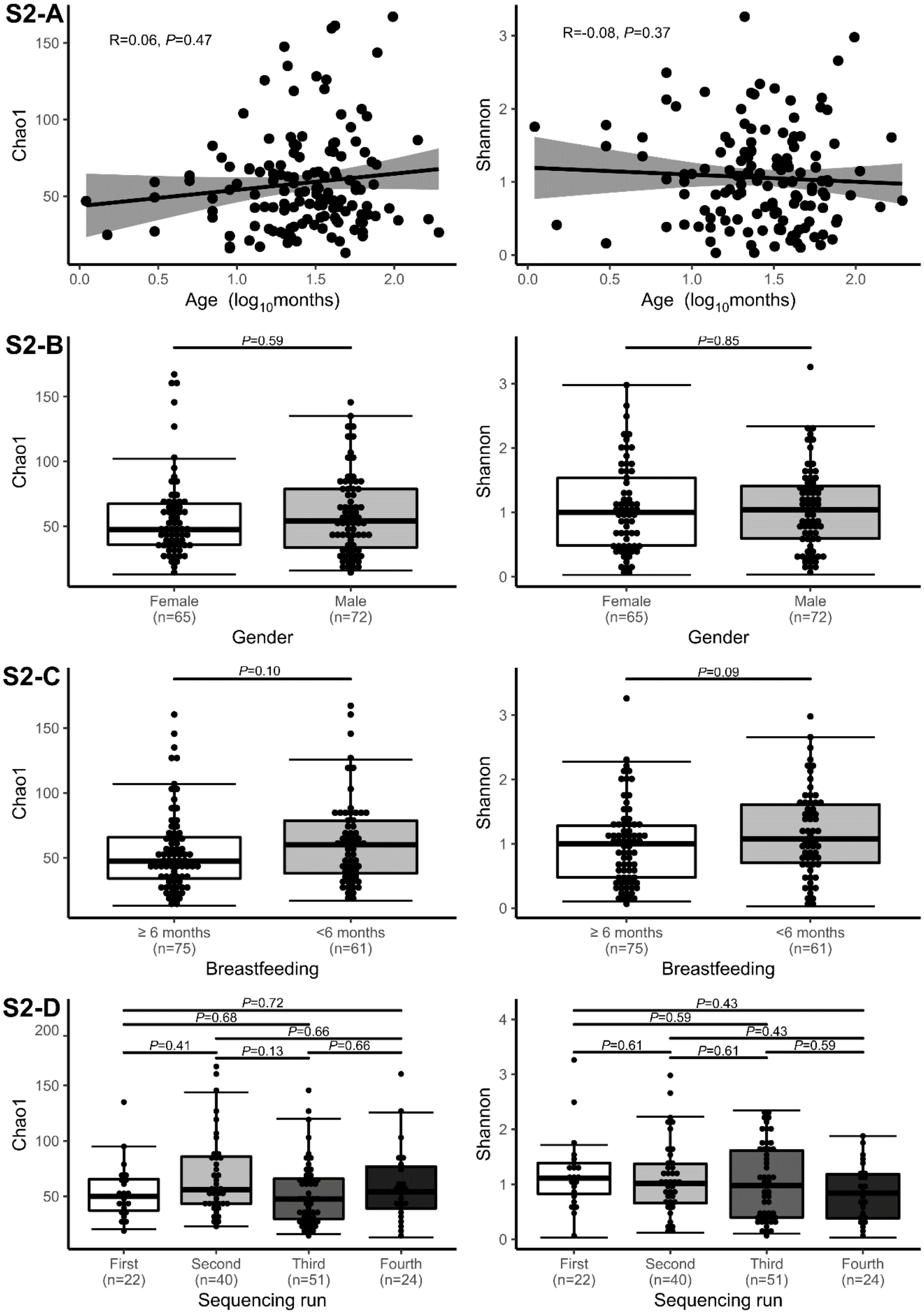

Figure S3. Principal Coordinate Analysis (PCoA) plot of the nasopharyngeal microbiota dissimilarities using a Bray-Curtis metric. Differences according to respiratory health status (S3-A), breastfeeding fulfillment of WHO recommendations (S3-B), vaccination status (S3-C), viral infection (S3-D), pneumococcal load (S3-E), age (S3-F), gender (S3-G), and sequencing run (S3-H) are shown.

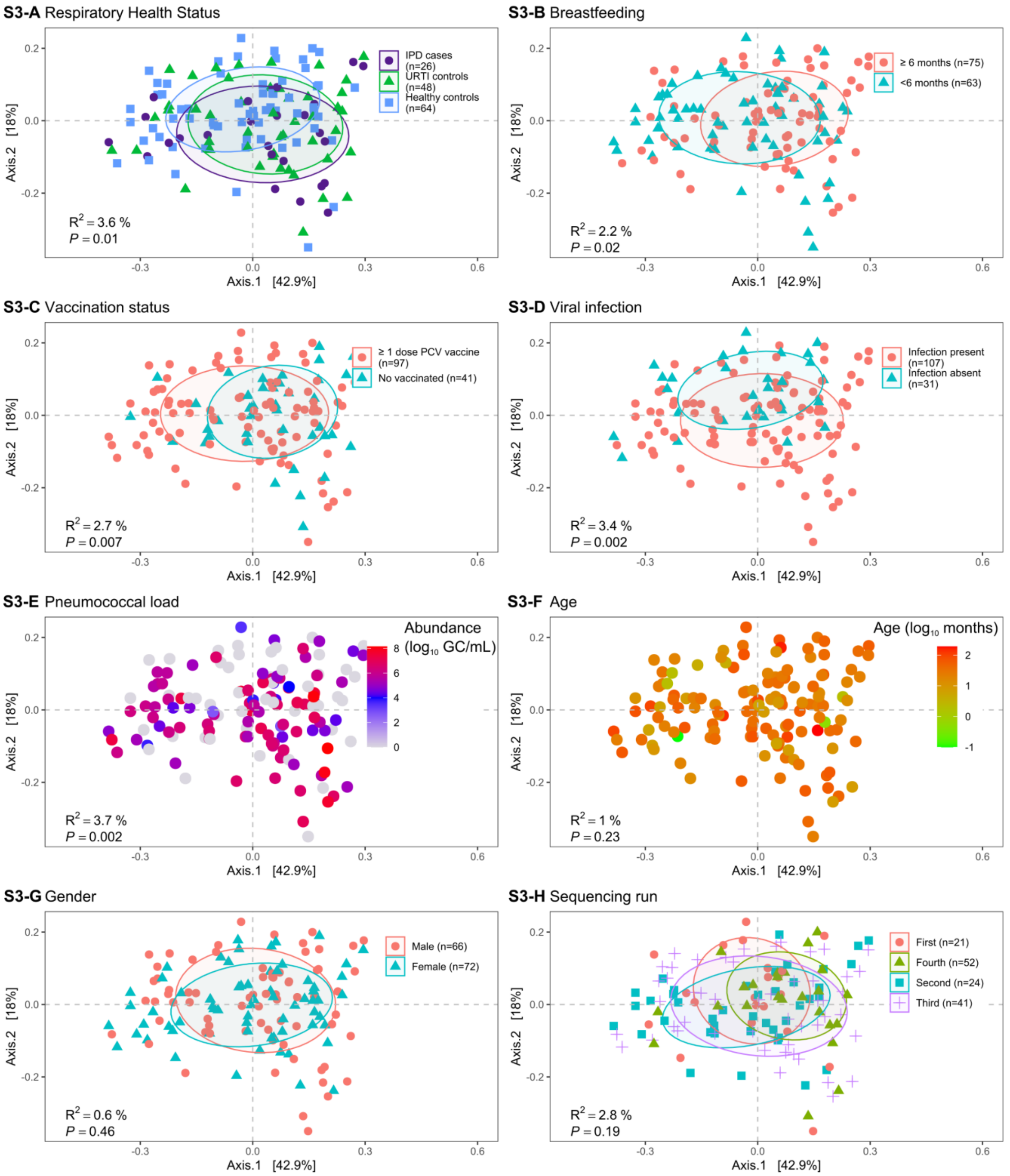

Figure S4. Principal Coordinate analyses (PCoA) plot of the nasopharyngeal microbiota dissimilarities using a Jaccard metric. Differences according to respiratory health status (S4-A), breastfeeding fulfillment of WHO recommendations (S4-B), vaccination status (S4-C), viral infection (S4-D), pneumococcal load (S4-E), age (S4-F), gender (S4-G), and sequencing run (S4-H) are shown.

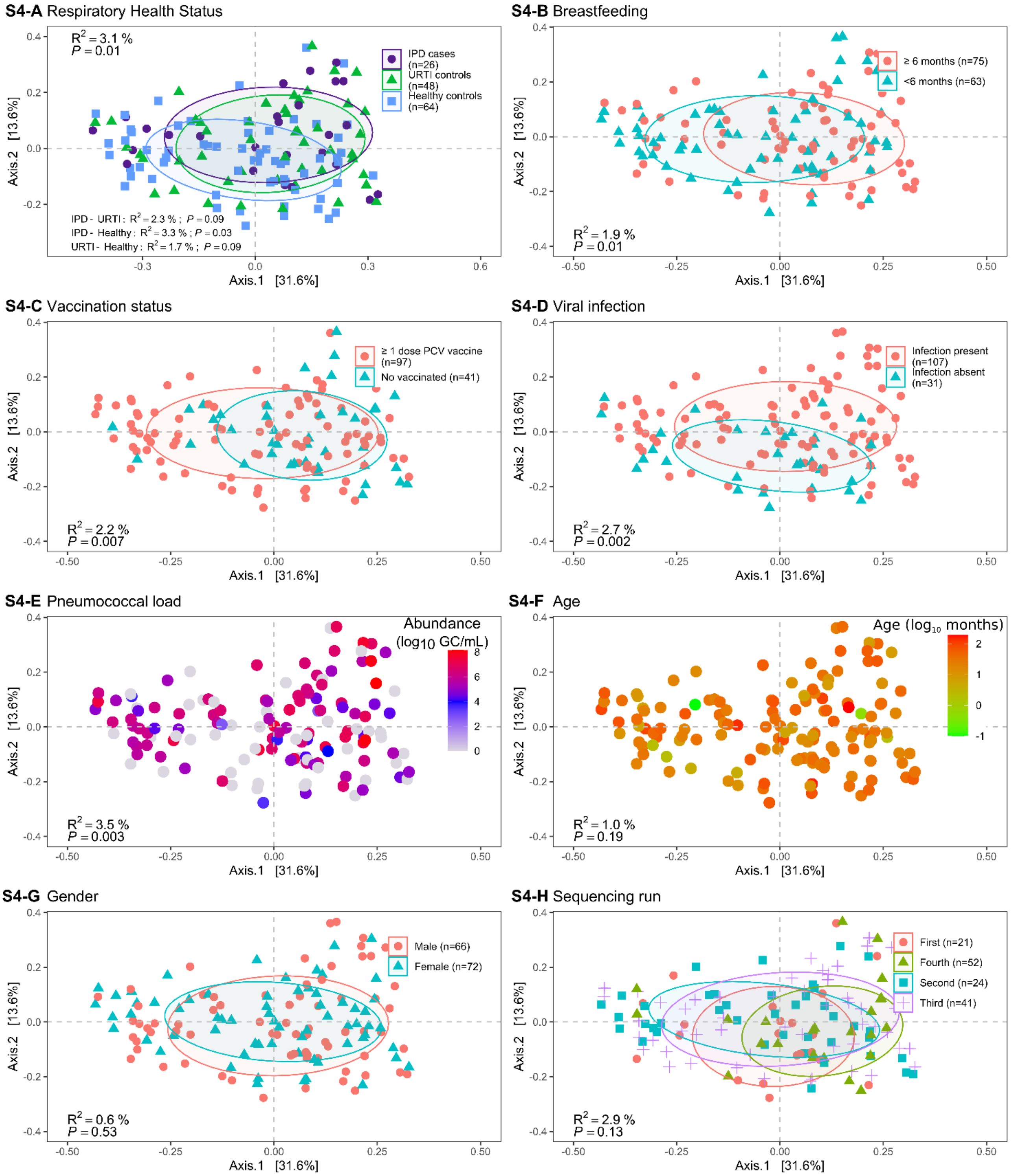

Figure S5. Principal Coordinate analyses (PCoA) plot of the nasopharyngeal microbiota dissimilarities using a Weighted Unifrac distance metric. Differences according to respiratory health status (S5-A), breastfeeding fulfillment of WHO recommendations (S5-B), vaccination status (S5-C), viral infection (S5-D), pneumococcal load (S5-E), age (S5-F), gender (S5-G), and sequencing run (S5-H) are shown.

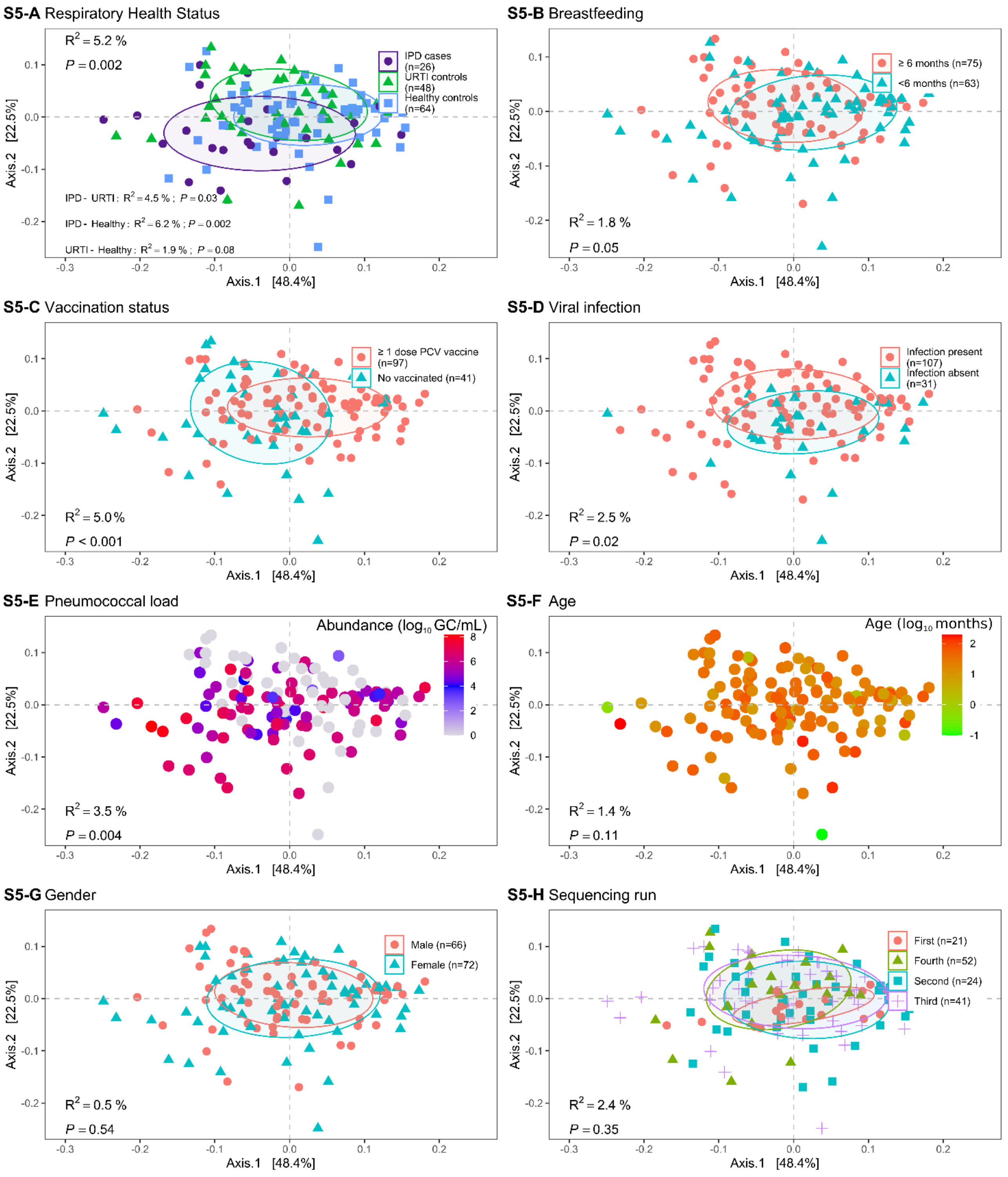

Figure S6. Relative abundances (RA) of genera according to respiratory health status. A RA heatmap of specific genera across samples is shown in (S6-A). Samples belonging to the same group were putted together in the *x* axis. In addition, LEfSe identified bacterial genera with statistically significant differences on their RA between IPD cases and URTI controls (S6-B), IPD cases and healthy controls (S6-C) and between both control groups (S6-D). OTUs were merged at the genus level.

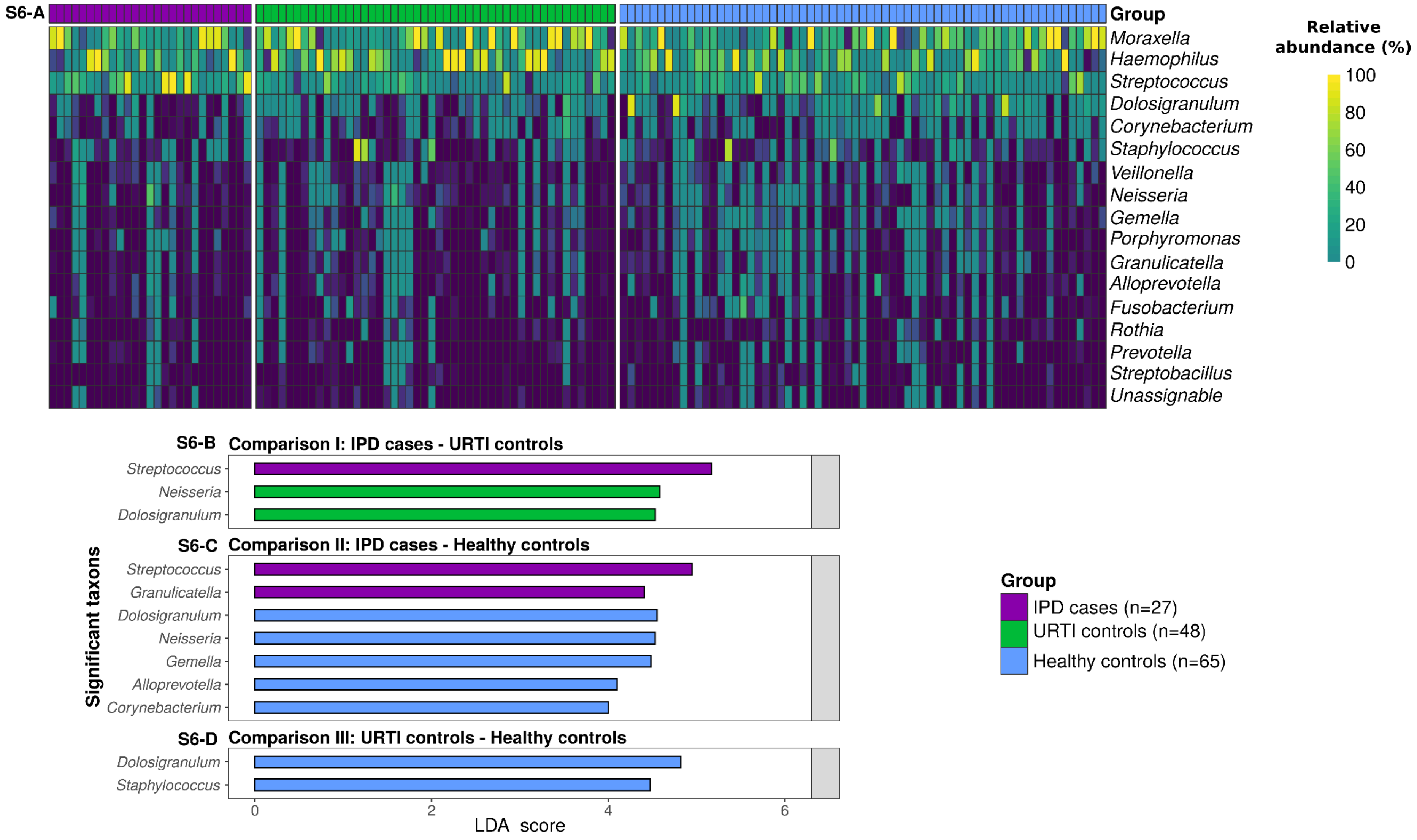

Figure S7. Taxa associated to different environmental variables. LEfSe identified bacterial taxons with statistically significant differences on their relative abundance according to vaccination status, breastfeeding fulfillment of WHO recommendations and viral infection. Such tests were performed at the genus (S7-A) and OTUs level (S7-B).

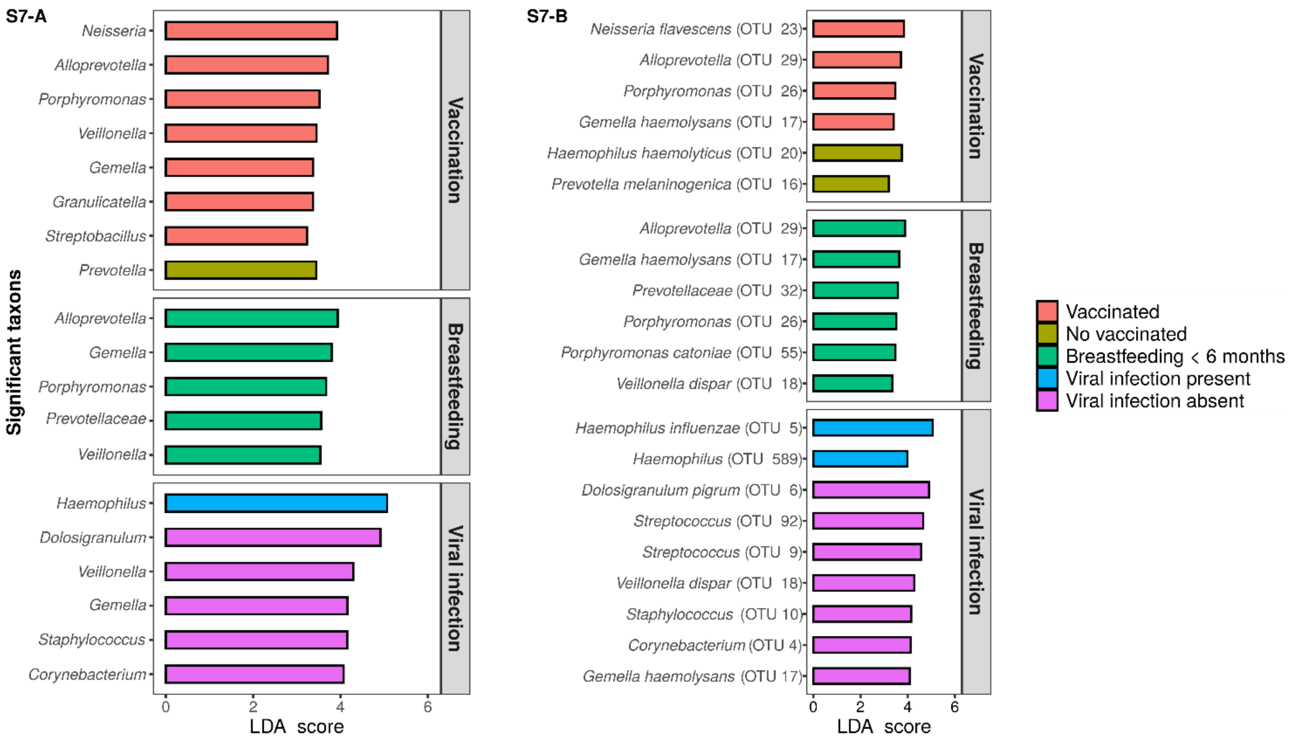

Figure S8. Performance of RF Classifier models with microbiota taxa at the OTUs level. Comparison of ROC curves of the different models is shown in (S8-A). In addition, the contribution of the variables to the performance of the 3 models is shown in (S8 B-C), through the representation of the Mean Decrease in Gini index of each variable.

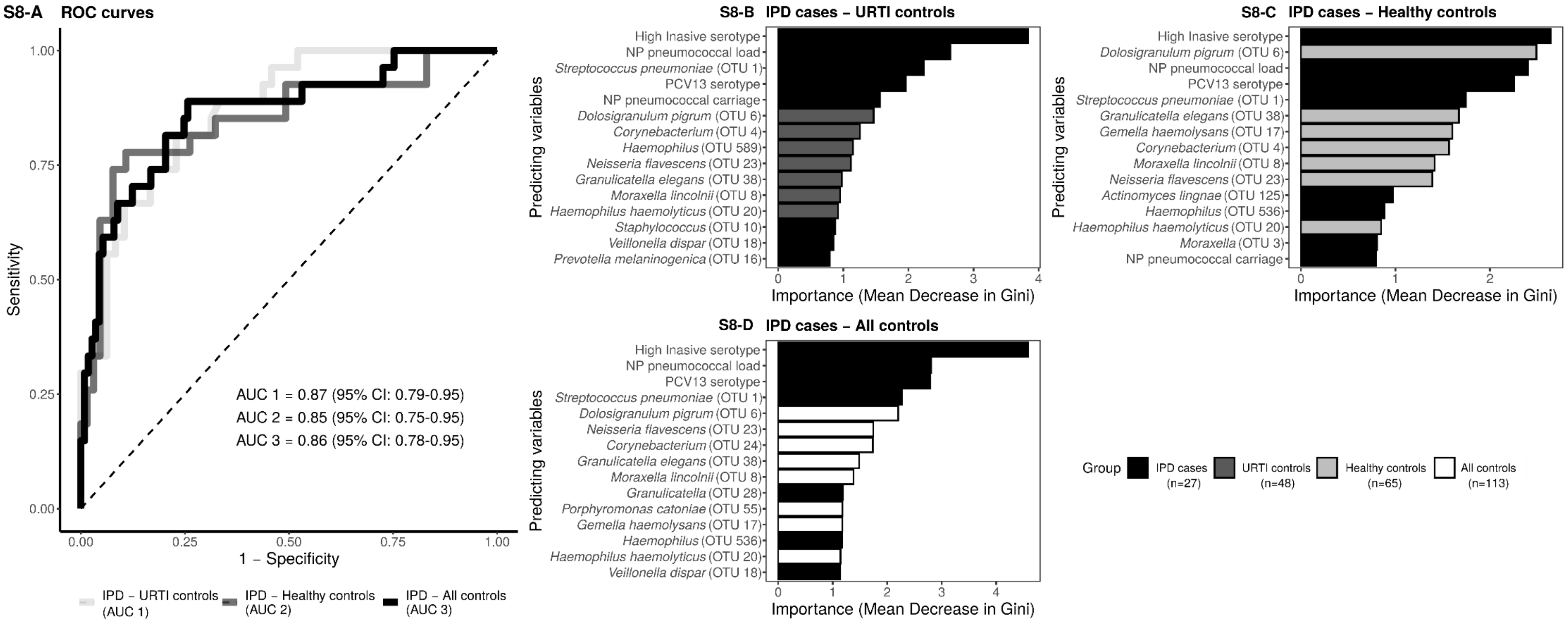

Table S1. OTU table clean-up. Summary of the statistics concerning number of sequences and OTUs obtained at each clean-up step.

	Samples and negative controls (n=156)				Samples (n=140)				Negative controls (n=16)			
	**Total (OTUs)**	**Total (seqs)**	**Median (seqs)**	**IQR (seqs)**	**Total (OTUs)**	**Total (seqs)**	**Median (seqs)**	**IQR (seqs)**	**Total (OTUs)**	**Total (seqs)**	**Median (seqs)**	**IQR (seqs)**
**After quality filtering and trimming**	726	11,769,440	69,353	50,673-94,333	706	11,753,943	73,589	56,455 - 95,936	189	15,497	764	397-1,224
**After ratio contaminant removal**	640	11,716,452	69,269	48,232-94,174	640	11,710,249	73,556	55,994 - 95,926	109	6,203	296	192-580
**After Decontam package**	602	1,701,218	69,078	47,933-93,457	602	11,696,096	73,553	55,974 - 95,915	77	5,122	244	150-463

Abbreviations. **OTU**: Operational Taxonomic Unit; **seqs**: Sequences, **IQR**; Inter-Quartile Range, **RA**: Relative Abundance.

Table S2. Median relative abundances of genera. Table containing median relative abundances of genera according to respiratory health status.

Genus	IPD cases (n=27)	URTI controls (n=48)	Healthy controls (n=65)
	Median (%)	Median (%)	Median (%)
Streptococcus	26.30	2.57	6.41
Moraxella	19.99	22.47	27.58
Haemophilus	6.76	22.82	16.69
Veillonella	0.02	0.01	0.04
Staphylococcus	0.02	0.01	0.03
Granulicatella	0.01	0.01	0.02
Gemella	0.01	0.01	0.05
Corynebacterium	0.01	0.04	0.12
Dolosigranulum	0.01	0.21	1.06
Rothia	0.00	0.00	0.00
Streptobacillus	0.00	0.00	0.00
Unassignable	0.00	0.00	0.00
Prevotella	0.00	0.00	0.00
Neisseria	0.00	0.02	0.04
Porphyromonas	0.00	0.01	0.02
Fusobacterium	0.00	0.00	0.01
Alloprevotella	0.00	0.01	0.01

Table S3. Median relative abundances of OTUs. Table containing median relative abundances of OTUs according to respiratory health status.

OTUs	IPD cases (n=27)	URTI controls (n=48)	Healthy controls (n=65)
	Median (%)	Median (%)	Median (%)
Moraxella (OTU 3)	19.79	14.10	21.02
Haemophilus influenzae (OTU 5)	2.97	19.61	12.12
Streptococcus pneumoniae (OTU 1)	19.99	2.42	6.39
Dolosigranulum pigrum (OTU 6)	0.01	0.21	1.06
Corynebacterium (OTU 4)	0.01	0.04	0.12
Haemophilus haemolyticus (OTU 20)	0.03	0.05	0.09
Gemella haemolysans; (OTU 17)	0.01	0.01	0.05
Staphylococcus (OTU 10)	0.02	0.01	0.03
Haemophilus (OTU 536)	0.02	0.02	0.03
Neisseria flavescens (OTU 23)	0.00	0.01	0.02
Moraxella lincolnii (OTU 8)	0.00	0.01	0.01
Alloprevotella (OTU 29)	0.00	0.01	0.01
Fusobacterium (OTU 36)	0.00	0.00	0.01
*Haemophilus (*OTU 589)	0.01	0.06	0.01
Porphyromonas (OTU 26)	0.00	0.00	0.01
Veillonella dispar (OTU 18)	0.01	0.00	0.01
Streptococcus (OTU 92)	0.01	0.01	0.01
Veillonella (OTU 30)	0.00	0.00	0.01
Streptococcus (OTU 9)	0.01	0.00	0.01
Granulicatella elegans (OTU 38)	0.00	0.01	0.01
Porphyromonas catoniae (OTU 55)	0.00	0.00	0.01
*Prevotella melaninogenica (*OTU 16)	0.00	0.00	0.00
Prevotellaceae (OTU 32)	0.00	0.00	0.00
Haemophilus parainfluenzae (OTU 31)	0.00	0.00	0.00
Streptobacillus moniliformis (OTU 78)	0.00	0.00	0.00
Moraxella nonliquefaciens (OTU 571)	0.00	0.00	0.00
Veillonella atypica (OTU 59)	0.00	0.00	0.00
Rothia mucilaginosa (OTU 35)	0.00	0.00	0.00
Neisseria (OTU 34)	0.00	0.00	0.00
Granulicatella (OTU 28)	0.00	0.00	0.00

Abbreviations. **OTU**: Operational Taxonomic Unit.

Table S4. Bacterial correlations at the OTUs level. Spearman's correlation coefficients and associated *P*-values for correlations between all OTUs detected in the nasopharyngeal samples from the children under study. All significant and non-significant bacterial interrelations are shown.

Taxon 1	Taxon 2	Correlation coefficient	*P*-value
Streptococcus pneumoniae (OTU 1)	*Staphylococcus (OTU 10)*	0.147	0.084
Streptococcus pneumoniae (OTU 1)	*Granulicatella (OTU 28)*	0.227	0.007
Staphylococcus (OTU 10)	*Granulicatella (OTU 28)*	0.271	0.001
Streptococcus pneumoniae (OTU 1)	*Dolosigranulum pigrum (OTU 6)*	-0.140	0.099
Staphylococcus (OTU 10)	*Dolosigranulum pigrum (OTU 6)*	0.286	<0.001
Granulicatella (OTU 28)	*Dolosigranulum pigrum (OTU 6)*	0.049	0.564
Streptococcus pneumoniae (OTU 1)	*Streptococcus (OTU 9)*	0.183	0.030
Staphylococcus (OTU 10)	*Streptococcus (OTU 9)*	0.393	<0.001
Granulicatella (OTU 28)	*Streptococcus (OTU 9)*	0.522	<0.001
Dolosigranulum pigrum (OTU 6)	*Streptococcus (OTU 9)*	0.200	0.018
Streptococcus pneumoniae (OTU 1)	*Veillonella (OTU 30)*	0.216	0.010
Staphylococcus (OTU 10)	*Veillonella (OTU 30)*	0.119	0.160
Granulicatella (OTU 28)	*Veillonella (OTU 30)*	0.552	<0.001
Dolosigranulum pigrum (OTU 6)	*Veillonella (OTU 30)*	-0.106	0.213
Streptococcus (OTU 9)	*Veillonella (OTU 30)*	0.394	<0.001
Streptococcus pneumoniae (OTU 1)	*Veillonella dispar (OTU 18)*	0.313	<0.001
Staphylococcus (OTU 10)	*Veillonella dispar (OTU 18)*	0.390	<0.001
Granulicatella (OTU 28)	*Veillonella dispar (OTU 18)*	0.625	<0.001
Dolosigranulum pigrum (OTU 6)	*Veillonella dispar (OTU 18)*	-0.015	0.862
Streptococcus (OTU 9)	*Veillonella dispar (OTU 18)*	0.610	<0.001
Veillonella (OTU 30)	*Veillonella dispar (OTU 18)*	0.559	<0.001
Streptococcus pneumoniae (OTU 1)	*Veillonella atypica OTU 59*	0.198	0.019
Staphylococcus (OTU 10)	*Veillonella atypica (OTU 59)*	0.175	0.038
Granulicatella (OTU 28)	*Veillonella atypica (OTU 59)*	0.573	<0.001
Dolosigranulum pigrum (OTU 6)	*Veillonella atypica (OTU 59)*	0.075	0.381

Streptococcus (OTU 9)	*Veillonella atypica (OTU 59)*	0.465	<0.001
Veillonella (OTU 30)	*Veillonella atypica (OTU 59)*	0.387	<0.001
Veillonella dispar (OTU 18)	*Veillonella atypica (OTU 59)*	0.551	<0.001
Streptococcus pneumoniae (OTU 1)	*Streptococcus (OTU 92)*	0.369	<0.001
Staphylococcus (OTU 10)	*Streptococcus (OTU 92)*	0.407	<0.001
Granulicatella (OTU 28)	*Streptococcus (OTU 92)*	0.644	<0.001
Dolosigranulum pigrum (OTU 6)	*Streptococcus (OTU 92)*	-0.003	0.971
Streptococcus (OTU 9)	*Streptococcus (OTU 92)*	0.572	<0.001
Veillonella (OTU 30)	*Streptococcus (OTU 92)*	0.546	<0.001
Veillonella dispar (OTU 18)	*Streptococcus (OTU 92)*	0.663	<0.001
Veillonella atypica (OTU 59)	*Streptococcus (OTU 92)*	0.490	<0.001
Streptococcus pneumoniae (OTU 1)	*Haemophilus (OTU 589)*	-0.080	0.345
Staphylococcus (OTU 10)	*Haemophilus (OTU 589)*	-0.338	<0.001
Granulicatella (OTU 28)	*Haemophilus (OTU 589)*	-0.016	0.850
Dolosigranulum pigrum (OTU 6)	*Haemophilus (OTU 589)*	-0.186	0.028
Streptococcus (OTU 9)	*Haemophilus (OTU 589)*	-0.229	0.007
Veillonella (OTU 30)	*Haemophilus (OTU 589)*	-0.021	0.806
Veillonella dispar (OTU 18)	*Haemophilus (OTU 589)*	-0.253	0.003
Veillonella atypica (OTU 59)	*Haemophilus (OTU 589)*	-0.147	0.082
Streptococcus (OTU 92)	*Haemophilus (OTU 589)*	-0.189	0.025
Streptococcus pneumoniae (OTU 1)	*Haemophilus (OTU 536)*	0.058	0.495
Staphylococcus (OTU 10)	*Haemophilus (OTU 536)*	-0.198	0.019
Granulicatella (OTU 28)	*Haemophilus (OTU 536)*	0.044	0.602
Dolosigranulum pigrum (OTU 6)	*Haemophilus (OTU 536)*	-0.185	0.029
Streptococcus (OTU 9)	*Haemophilus (OTU 536)*	-0.201	0.017
Veillonella (OTU 30)	*Haemophilus (OTU 536)*	0.160	0.059
Veillonella dispar (OTU 18)	*Haemophilus (OTU 536)*	0.003	0.972
Veillonella atypica (OTU 59)	*Haemophilus (OTU 536)*	-0.016	0.849
Streptococcus (OTU 92)	*Haemophilus (OTU 536)*	-0.011	0.897
Haemophilus (OTU 589)	*Haemophilus (OTU 536)*	0.546	<0.001
Streptococcus pneumoniae (OTU 1)	*Haemophilus haemolyticus (OTU 20)*	0.181	0.032
Staphylococcus (OTU 10)	*Haemophilus haemolyticus (OTU 20)*	-0.066	0.436
Granulicatella (OTU 28)	*Haemophilus haemolyticus (OTU 20)*	0.334	<0.001

Dolosigranulum pigrum (OTU 6)	*Haemophilus haemolyticus (OTU 20)*	-0.166	0.050
Streptococcus (OTU 9)	*Haemophilus haemolyticus (OTU 20)*	0.076	0.374
Veillonella (OTU 30)	*Haemophilus haemolyticus (OTU 20)*	0.390	<0.001
Veillonella dispar (OTU 18)	*Haemophilus haemolyticus (OTU 20)*	0.292	<0.001
Veillonella atypica (OTU 59)	*Haemophilus haemolyticus (OTU 20)*	0.255	0.002
Streptococcus (OTU 92)	*Haemophilus haemolyticus (OTU 20)*	0.280	<0.001
Haemophilus (OTU 589)	*Haemophilus haemolyticus (OTU 20)*	0.314	<0.001
Haemophilus (OTU 536)	*Haemophilus haemolyticus (OTU 20)*	0.701	<0.001
Streptococcus pneumoniae (OTU 1)	*Fusobacterium ((OTU 36))*	0.208	0.013
Staphylococcus (OTU 10)	*Fusobacterium (OTU 36)*	0.158	0.062
Granulicatella (OTU 28)	*Fusobacterium (OTU 36)*	0.493	<0.001
Dolosigranulum pigrum (OTU 6)	*Fusobacterium (OTU 36)*	-0.078	0.359
Streptococcus (OTU 9)	*Fusobacterium (OTU 36)*	0.349	<0.001
Veillonella (OTU 30)	*Fusobacterium (OTU 36)*	0.485	<0.001
Veillonella dispar (OTU 18)	*Fusobacterium (OTU 36)*	0.435	<0.001
Veillonella atypica (OTU 59)	*Fusobacterium (OTU 36)*	0.356	<0.001
Streptococcus (OTU 92)	*Fusobacterium (OTU 36)*	0.409	<0.001
Haemophilus (OTU 589)	*Fusobacterium (OTU 36)*	-0.025	0.768
Haemophilus (OTU 536)	*Fusobacterium (OTU 36)*	0.141	0.096
Haemophilus haemolyticus (OTU 20)	*Fusobacterium (OTU 36)*	0.269	0.001
Streptococcus pneumoniae (OTU 1)	*Streptobacillus moniliformis (OTU 78)*	0.259	0.002
Staphylococcus (OTU 10)	*Streptobacillus moniliformis (OTU 78)*	-0.057	0.500
Granulicatella (OTU 28)	*Streptobacillus moniliformis (OTU 78)*	0.329	<0.001
Dolosigranulum pigrum (OTU 6)	*Streptobacillus moniliformis (OTU 78)*	-0.098	0.251
Streptococcus (OTU 9)	*Streptobacillus moniliformis (OTU 78)*	0.240	0.004
Veillonella (OTU 30)	*Streptobacillus moniliformis (OTU 78)*	0.512	<0.001
Veillonella dispar (OTU 18)	*Streptobacillus moniliformis (OTU 78)*	0.269	0.001
Veillonella atypica (OTU 59)	*Streptobacillus moniliformis (OTU 78)*	0.222	0.008
Streptococcus (OTU 92)	*Streptobacillus moniliformis (OTU 78)*	0.326	<0.001
Haemophilus (OTU 589)	*Streptobacillus moniliformis (OTU 78)*	0.000	0.998
Haemophilus (OTU 536)	*Streptobacillus moniliformis (OTU 78)*	0.101	0.236
Haemophilus haemolyticus (OTU 20)	*Streptobacillus moniliformis (OTU 78)*	0.270	0.001
Fusobacterium (OTU 36)	*Streptobacillus moniliformis (OTU 78)*	0.421	<0.001

Streptococcus pneumoniae (OTU 1)	*Alloprevotella (OTU 29)*	0.292	<0.001
Staphylococcus (OTU 10)	*Alloprevotella (OTU 29)*	0.149	0.079
Granulicatella (OTU 28)	*Alloprevotella (OTU 29)*	0.575	<0.001
Dolosigranulum pigrum (OTU 6)	*Alloprevotella (OTU 29)*	-0.007	0.937
Streptococcus (OTU 9)	*Alloprevotella (OTU 29)*	0.390	<0.001
Veillonella (OTU 30)	*Alloprevotella (OTU 29)*	0.708	<0.001
Veillonella dispar (OTU 18)	*Alloprevotella (OTU 29)*	0.530	<0.001
Veillonella atypica (OTU 59)	*Alloprevotella (OTU 29)*	0.350	<0.001
Streptococcus (OTU 92)	*Alloprevotella (OTU 29)*	0.500	<0.001
Haemophilus (OTU 589)	*Alloprevotella (OTU 29)*	-0.068	0.423
Haemophilus (OTU 536)	*Alloprevotella (OTU 29)*	0.143	0.092
Haemophilus haemolyticus (OTU 20)	*Alloprevotella (OTU 29)*	0.398	<0.001
Fusobacterium (OTU 36)	*Alloprevotella (OTU 29)*	0.532	<0.001
Streptobacillus moniliformis (OTU 78)	*Alloprevotella (OTU 29)*	0.502	<0.001
Streptococcus pneumoniae (OTU 1)	*Unassignable (OTU 32)*	0.192	0.023
Staphylococcus (OTU 10)	*Unassignable (OTU 32)*	0.147	0.083
Granulicatella (OTU 28)	*Unassignable (OTU 32)*	0.493	<0.001
Dolosigranulum pigrum (OTU 6)	*Unassignable (OTU 32)*	-0.040	0.638
Streptococcus (OTU 9)	*Unassignable (OTU 32)*	0.438	<0.001
Veillonella (OTU 30)	*Unassignable (OTU 32)*	0.563	<0.001
Veillonella dispar (OTU 18)	*Unassignable (OTU 32)*	0.527	<0.001
Veillonella atypica (OTU 59)	*Unassignable (OTU 32)*	0.447	<0.001
Streptococcus (OTU 92)	*Unassignable (OTU 32)*	0.431	<0.001
Haemophilus (OTU 589)	*Unassignable (OTU 32)*	-0.080	0.349
Haemophilus (OTU 536)	*Unassignable (OTU 32)*	0.046	0.588
Haemophilus haemolyticus (OTU 20)	*Unassignable (OTU 32)*	0.283	<0.001
Fusobacterium (OTU 36)	*Unassignable (OTU 32)*	0.552	<0.001
Streptobacillus moniliformis (OTU 78)	*Unassignable (OTU 32)*	0.515	<0.001
Alloprevotella (OTU 29)	*Unassignable (OTU 32)*	0.622	<0.001
Streptococcus pneumoniae (OTU 1)	*Porphyromonas (OTU 26)*	0.197	0.019
Staphylococcus (OTU 10)	*Porphyromonas (OTU 26)*	0.103	0.227
Granulicatella (OTU 28)	*Porphyromonas (OTU 26)*	0.508	<0.001
Dolosigranulum pigrum (OTU 6)	*Porphyromonas (OTU 26)*	-0.075	0.381

Streptococcus (OTU 9)	*Porphyromonas (OTU 26)*	0.343	<0.001
Veillonella (OTU 30)	*Porphyromonas (OTU 26)*	0.712	<0.001
Veillonella dispar (OTU 18)	*Porphyromonas (OTU 26)*	0.534	<0.001
Veillonella atypica (OTU 59)	*Porphyromonas (OTU 26)*	0.473	<0.001
Streptococcus (OTU 92)	*Porphyromonas (OTU 26)*	0.420	<0.001
Haemophilus (OTU 589)	*Porphyromonas (OTU 26)*	-0.027	0.748
Haemophilus (OTU 536)	*Porphyromonas (OTU 26)*	0.213	0.012
Haemophilus haemolyticus (OTU 20)	*Porphyromonas (OTU 26)*	0.432	<0.001
Fusobacterium (OTU 36)	*Porphyromonas (OTU 26)*	0.474	<0.001
Streptobacillus moniliformis (OTU 78)	*Porphyromonas (OTU 26)*	0.504	<0.001
Alloprevotella (OTU 29)	*Porphyromonas (OTU 26)*	0.619	<0.001
Unassignable (OTU 32)	*Porphyromonas (OTU 26)*	0.616	<0.001
Streptococcus pneumoniae (OTU 1)	*Porphyromonas catoniae (OTU 55)*	0.250	0.003
Staphylococcus (OTU 10)	*Porphyromonas catoniae (OTU 55)*	0.119	0.160
Granulicatella (OTU 28)	*Porphyromonas catoniae (OTU 55)*	0.519	<0.001
Dolosigranulum pigrum (OTU 6)	*Porphyromonas catoniae (OTU 55)*	-0.131	0.124
Streptococcus (OTU 9)	*Porphyromonas catoniae (OTU 55)*	0.395	<0.001
Veillonella (OTU 30)	*Porphyromonas catoniae (OTU 55)*	0.626	<0.001
Veillonella dispar (OTU 18)	*Porphyromonas catoniae (OTU 55)*	0.499	<0.001
Veillonella atypica (OTU 59)	*Porphyromonas catoniae (OTU 55)*	0.409	<0.001
Streptococcus (OTU 92)	*Porphyromonas catoniae (OTU 55)*	0.477	<0.001
Haemophilus (OTU 589)	*Porphyromonas catoniae (OTU 55)*	-0.016	0.849
Haemophilus (OTU 536)	*Porphyromonas catoniae (OTU 55)*	0.181	0.032
Haemophilus haemolyticus (OTU 20)	*Porphyromonas catoniae (OTU 55)*	0.387	<0.001
Fusobacterium (OTU 36)	*Porphyromonas catoniae (OTU 55)*	0.503	<0.001
Streptobacillus moniliformis (OTU 78)	*Porphyromonas catoniae (OTU 55)*	0.517	<0.001
Alloprevotella (OTU 29)	*Porphyromonas catoniae (OTU 55)*	0.640	<0.001
Unassignable (OTU 32)	*Porphyromonas catoniae (OTU 55)*	0.627	<0.001
Porphyromonas (OTU 26)	*Porphyromonas catoniae (OTU 55)*	0.644	<0.001
Streptococcus pneumoniae (OTU 1)	*Prevotella melaninogenica (OTU 16)*	0.213	0.011
Staphylococcus (OTU 10)	*Prevotella melaninogenica (OTU 16)*	0.070	0.413
Granulicatella (OTU 28)	*Prevotella melaninogenica (OTU 16)*	0.582	<0.001
Dolosigranulum pigrum (OTU 6)	*Prevotella melaninogenica (OTU 16)*	-0.093	0.275

Streptococcus (OTU 9)	*Prevotella melaninogenica (OTU 16)*	0.412	<0.001
Veillonella (OTU 30)	*Prevotella melaninogenica (OTU 16)*	0.612	<0.001
Veillonella dispar (OTU 18)	*Prevotella melaninogenica (OTU 16)*	0.585	<0.001
Veillonella atypica (OTU 59)	*Prevotella melaninogenica (OTU 16)*	0.427	<0.001
Streptococcus (OTU 92)	*Prevotella melaninogenica (OTU 16)*	0.494	<0.001
Haemophilus (OTU 589)	*Prevotella melaninogenica (OTU 16)*	-0.037	0.662
Haemophilus (OTU 536)	*Prevotella melaninogenica (OTU 16)*	0.044	0.607
Haemophilus haemolyticus (OTU 20)	*Prevotella melaninogenica (OTU 16)*	0.296	<0.001
Fusobacterium (OTU 36)	*Prevotella melaninogenica (OTU 16)*	0.577	<0.001
Streptobacillus moniliformis (OTU 78)	*Prevotella melaninogenica (OTU 16)*	0.405	<0.001
Alloprevotella (OTU 29)	*Prevotella melaninogenica (OTU 16)*	0.548	<0.001
Unassignable (OTU 32)	*Prevotella melaninogenica (OTU 16)*	0.555	<0.001
Porphyromonas (OTU 26)	*Prevotella melaninogenica (OTU 16)*	0.557	<0.001
Porphyromonas catoniae (OTU 55)	*Prevotella melaninogenica (OTU 16)*	0.558	<0.001
Streptococcus pneumoniae (OTU 1)	*Neisseria flavescens (OTU 23)*	0.226	0.007
Staphylococcus (OTU 10)	*Neisseria flavescens (OTU 23)*	0.122	0.152
Granulicatella (OTU 28)	*Neisseria flavescens (OTU 23)*	0.571	<0.001
Dolosigranulum pigrum (OTU 6)	*Neisseria flavescens (OTU 23)*	0.004	0.967
Streptococcus (OTU 9)	*Neisseria flavescens (OTU 23)*	0.409	<0.001
Veillonella (OTU 30)	*Neisseria flavescens (OTU 23)*	0.571	<0.001
Veillonella dispar (OTU 18)	*Neisseria flavescens (OTU 23)*	0.531	<0.001
Veillonella atypica (OTU 59)	*Neisseria flavescens (OTU 23)*	0.398	<0.001
Streptococcus (OTU 92)	*Neisseria flavescens (OTU 23)*	0.449	<0.001
Haemophilus (OTU 589)	*Neisseria flavescens (OTU 23)*	0.006	0.942
Haemophilus (OTU 536)	*Neisseria flavescens (OTU 23)*	0.173	0.041
Haemophilus haemolyticus (OTU 20)	*Neisseria flavescens (OTU 23)*	0.416	<0.001
Fusobacterium (OTU 36)	*Neisseria flavescens (OTU 23)*	0.531	<0.001
Streptobacillus moniliformis (OTU 78)	*Neisseria flavescens (OTU 23)*	0.526	<0.001
Alloprevotella (OTU 29)	*Neisseria flavescens (OTU 23)*	0.662	<0.001
Unassignable (OTU 32)	*Neisseria flavescens (OTU 23)*	0.573	<0.001
Porphyromonas (OTU 26)	*Neisseria flavescens (OTU 23)*	0.625	<0.001
Porphyromonas catoniae (OTU 55)	*Neisseria flavescens (OTU 23)*	0.605	<0.001
Prevotella melaninogenica (OTU 16)	*Neisseria flavescens (OTU 23)*	0.496	<0.001

Streptococcus pneumoniae (OTU 1)	*Moraxella (OTU 3)*	-0.127	0.134
Staphylococcus (OTU 10)	*Moraxella (OTU 3)*	-0.026	0.765
Granulicatella (OTU 28)	*Moraxella (OTU 3)*	-0.183	0.030
Dolosigranulum pigrum (OTU 6)	*Moraxella (OTU 3)*	0.198	0.019
Streptococcus (OTU 9)	*Moraxella (OTU 3)*	0.055	0.520
Veillonella (OTU 30)	*Moraxella (OTU 3)*	-0.229	0.006
Veillonella dispar (OTU 18)	*Moraxella (OTU 3)*	-0.106	0.211
Veillonella atypica (OTU 59)	*Moraxella (OTU 3)*	-0.012	0.888
Streptococcus (OTU 92)	*Moraxella (OTU 3)*	-0.097	0.255
Haemophilus (OTU 589)	*Moraxella (OTU 3)*	-0.234	0.005
Haemophilus (OTU 536)	*Moraxella (OTU 3)*	-0.268	0.001
Haemophilus haemolyticus (OTU 20)	*Moraxella (OTU 3)*	-0.334	<0.001
Fusobacterium (OTU 36)	*Moraxella (OTU 3)*	-0.132	0.119
Streptobacillus moniliformis (OTU 78)	*Moraxella (OTU 3)*	-0.212	0.012
Alloprevotella (OTU 29)	*Moraxella (OTU 3)*	-0.231	0.006
Unassignable (OTU 32)	*Moraxella (OTU 3)*	-0.195	0.021
Porphyromonas (OTU 26)	*Moraxella (OTU 3)*	-0.135	0.112
Porphyromonas catoniae (OTU 55)	*Moraxella (OTU 3)*	-0.199	0.018
Prevotella melaninogenica (OTU 16)	*Moraxella (OTU 3)*	-0.262	0.002
Neisseria flavescens (OTU 23)	*Moraxella (OTU 3)*	-0.037	0.662
Streptococcus pneumoniae (OTU 1)	*Haemophilus parainfluenzae (OTU 31)*	0.174	0.040
Staphylococcus (OTU 10)	*Haemophilus parainfluenzae (OTU 31)*	0.357	<0.001
Granulicatella (OTU 28)	*Haemophilus parainfluenzae (OTU 31)*	0.717	<0.001
Dolosigranulum pigrum (OTU 6)	*Haemophilus parainfluenzae (OTU 31)*	0.032	0.712
Streptococcus (OTU 9)	*Haemophilus parainfluenzae (OTU 31)*	0.471	<0.001
Veillonella (OTU 30)	*Haemophilus parainfluenzae (OTU 31)*	0.554	<0.001
Veillonella dispar (OTU 18)	*Haemophilus parainfluenzae (OTU 31)*	0.706	<0.001
Veillonella atypica (OTU 59)	*Haemophilus parainfluenzae (OTU 31)*	0.477	<0.001
Streptococcus (OTU 92)	*Haemophilus parainfluenzae (OTU 31)*	0.681	<0.001
Haemophilus (OTU 589)	*Haemophilus parainfluenzae (OTU 31)*	-0.085	0.316
Haemophilus (OTU 536)	*Haemophilus parainfluenzae (OTU 31)*	0.110	0.197
Haemophilus haemolyticus (OTU 20)	*Haemophilus parainfluenzae (OTU 31)*	0.340	<0.001
Fusobacterium (OTU 36)	*Haemophilus parainfluenzae (OTU 31)*	0.533	<0.001

Streptobacillus moniliformis (OTU 78)	*Haemophilus parainfluenzae (OTU 31)*	0.343	<0.001
Alloprevotella (OTU 29)	*Haemophilus parainfluenzae (OTU 31)*	0.656	<0.001
Unassignable (OTU 32)	*Haemophilus parainfluenzae (OTU 31)*	0.528	<0.001
Porphyromonas (OTU 26)	*Haemophilus parainfluenzae (OTU 31)*	0.512	<0.001
Porphyromonas catoniae (OTU 55)	*Haemophilus parainfluenzae (OTU 31)*	0.578	<0.001
Prevotella melaninogenica (OTU 16)	*Haemophilus parainfluenzae (OTU 31)*	0.506	<0.001
Neisseria flavescens (OTU 23)	*Haemophilus parainfluenzae (OTU 31)*	0.625	<0.001
Moraxella (OTU 3)	*Haemophilus parainfluenzae (OTU 31)*	-0.196	0.020
Streptococcus pneumoniae (OTU 1)	*Neisseria (OTU 34)*	0.293	<0.001
Staphylococcus (OTU 10)	*Neisseria (OTU 34)*	0.319	<0.001
Granulicatella (OTU 28)	*Neisseria (OTU 34)*	0.526	<0.001
Dolosigranulum pigrum (OTU 6)	*Neisseria (OTU 34)*	0.033	0.699
Streptococcus (OTU 9)	*Neisseria (OTU 34)*	0.427	<0.001
Veillonella (OTU 30)	*Neisseria (OTU 34)*	0.503	<0.001
Veillonella dispar (OTU 18)	*Neisseria (OTU 34)*	0.531	<0.001
Veillonella atypica (OTU 59)	*Neisseria (OTU 34)*	0.307	<0.001
Streptococcus (OTU 92)	*Neisseria (OTU 34)*	0.552	<0.001
Haemophilus (OTU 589)	*Neisseria (OTU 34)*	-0.132	0.119
Haemophilus (OTU 536)	*Neisseria (OTU 34)*	0.027	0.752
Haemophilus haemolyticus (OTU 20)	*Neisseria (OTU 34)*	0.288	<0.001
Fusobacterium (OTU 36)	*Neisseria (OTU 34)*	0.433	<0.001
Streptobacillus moniliformis (OTU 78)	*Neisseria (OTU 34)*	0.340	<0.001
Alloprevotella (OTU 29)	*Neisseria (OTU 34)*	0.570	<0.001
Unassignable (OTU 32)	*Neisseria (OTU 34)*	0.471	<0.001
Porphyromonas (OTU 26)	*Neisseria (OTU 34)*	0.546	<0.001
Porphyromonas catoniae (OTU 55)	*Neisseria (OTU 34)*	0.544	<0.001
Prevotella melaninogenica (OTU 16)	*Neisseria (OTU 34)*	0.445	<0.001
Neisseria flavescens (OTU 23)	*Neisseria (OTU 34)*	0.568	<0.001
Moraxella (OTU 3)	*Neisseria (OTU 34)*	0.000	0.998
Haemophilus parainfluenzae (OTU 31)	*Neisseria (OTU 34)*	0.659	<0.001
Streptococcus pneumoniae (OTU 1)	*Rothia mucilaginosa (OTU 35)*	0.240	0.004
Staphylococcus (OTU 10)	*Rothia mucilaginosa (OTU 35)*	0.220	0.009
Granulicatella (OTU 28)	*Rothia mucilaginosa (OTU 35)*	0.496	<0.001

Dolosigranulum pigrum (OTU 6)	*Rothia mucilaginosa (OTU 35)*	-0.074	0.383
Streptococcus (OTU 9)	*Rothia mucilaginosa (OTU 35)*	0.498	<0.001
Veillonella (OTU 30)	*Rothia mucilaginosa (OTU 35)*	0.499	<0.001
Veillonella dispar (OTU 18)	*Rothia mucilaginosa (OTU 35)*	0.636	<0.001
Veillonella atypica (OTU 59)	*Rothia mucilaginosa (OTU 35)*	0.425	<0.001
Streptococcus (OTU 92)	*Rothia mucilaginosa (OTU 35)*	0.588	<0.001
Haemophilus (OTU 589)	*Rothia mucilaginosa (OTU 35)*	-0.161	0.058
Haemophilus (OTU 536)	*Rothia mucilaginosa (OTU 35)*	-0.076	0.372
Haemophilus haemolyticus (OTU 20)	*Rothia mucilaginosa (OTU 35)*	0.206	0.014
Fusobacterium (OTU 36)	*Rothia mucilaginosa (OTU 35)*	0.375	<0.001
Streptobacillus moniliformis (OTU 78)	*Rothia mucilaginosa (OTU 35)*	0.328	<0.001
Alloprevotella (OTU 29)	*Rothia mucilaginosa (OTU 35)*	0.389	<0.001
Unassignable (OTU 32)	*Rothia mucilaginosa (OTU 35)*	0.458	<0.001
Porphyromonas (OTU 26)	*Rothia mucilaginosa (OTU 35)*	0.465	<0.001
Porphyromonas catoniae (OTU 55)	*Rothia mucilaginosa (OTU 35)*	0.457	<0.001
Prevotella melaninogenica (OTU 16)	*Rothia mucilaginosa (OTU 35)*	0.531	<0.001
Neisseria flavescens (OTU 23)	*Rothia mucilaginosa (OTU 35)*	0.516	<0.001
Moraxella (OTU 3)	*Rothia mucilaginosa (OTU 35)*	-0.005	0.951
Haemophilus parainfluenzae (OTU 31)	*Rothia mucilaginosa (OTU 35)*	0.450	<0.001
Neisseria (OTU 34)	*Rothia mucilaginosa (OTU 35)*	0.408	<0.001
Streptococcus pneumoniae (OTU 1)	*Corynebacterium (OTU 4)*	-0.174	0.039
Staphylococcus (OTU 10)	*Corynebacterium (OTU 4)*	0.126	0.138
Granulicatella (OTU 28)	*Corynebacterium (OTU 4)*	0.007	0.939
Dolosigranulum pigrum (OTU 6)	*Corynebacterium (OTU 4)*	0.751	<0.001
Streptococcus (OTU 9)	*Corynebacterium (OTU 4)*	0.080	0.349
Veillonella (OTU 30)	*Corynebacterium (OTU 4)*	-0.131	0.124
Veillonella dispar (OTU 18)	*Corynebacterium (OTU 4)*	-0.082	0.333
Veillonella atypica (OTU 59)	*Corynebacterium (OTU 4)*	0.057	0.507
Streptococcus (OTU 92)	*Corynebacterium (OTU 4)*	-0.091	0.287
Haemophilus (OTU 589)	*Corynebacterium (OTU 4)*	-0.145	0.088
Haemophilus (OTU 536)	*Corynebacterium (OTU 4)*	-0.176	0.038
Haemophilus haemolyticus (OTU 20)	*Corynebacterium (OTU 4)*	-0.126	0.139
Fusobacterium (OTU 36)	*Corynebacterium (OTU 4)*	-0.053	0.534

Streptobacillus moniliformis (OTU 78)	*Corynebacterium (OTU 4)*	-0.031	0.715
Alloprevotella (OTU 29)	*Corynebacterium (OTU 4)*	-0.072	0.395
Unassignable (OTU 32)	*Corynebacterium (OTU 4)*	-0.072	0.398
Porphyromonas (OTU 26)	*Corynebacterium (OTU 4)*	-0.115	0.178
Porphyromonas catoniae (OTU 55)	*Corynebacterium (OTU 4)*	-0.164	0.053
Prevotella melaninogenica (OTU 16)	*Corynebacterium (OTU 4)*	-0.063	0.458
Neisseria flavescens (OTU 23)	*Corynebacterium (OTU 4)*	-0.060	0.478
Moraxella (OTU 3)	*Corynebacterium (OTU 4)*	0.096	0.257
Haemophilus parainfluenzae (OTU 31)	*Corynebacterium (OTU 4)*	-0.070	0.412
Neisseria (OTU 34)	*Corynebacterium (OTU 4)*	-0.048	0.577
Rothia mucilaginosa (OTU 35)	*Corynebacterium (OTU 4)*	-0.103	0.226
Streptococcus pneumoniae (OTU 1)	*Haemophilus influenzae (OTU 5)*	0.003	0.972
Staphylococcus (OTU 10)	*Haemophilus influenzae (OTU 5)*	-0.340	<0.001
Granulicatella (OTU 28)	*Haemophilus influenzae (OTU 5)*	-0.014	0.870
Dolosigranulum pigrum (OTU 6)	*Haemophilus influenzae (OTU 5)*	-0.226	0.007
Streptococcus (OTU 9)	*Haemophilus influenzae (OTU 5)*	-0.316	<0.001
Veillonella (OTU 30)	*Haemophilus influenzae (OTU 5)*	0.062	0.470
Veillonella dispar (OTU 18)	*Haemophilus influenzae (OTU 5)*	-0.202	0.017
Veillonella atypica (OTU 59)	*Haemophilus influenzae (OTU 5)*	-0.044	0.602
Streptococcus (OTU 92)	*Haemophilus influenzae (OTU 5)*	-0.088	0.304
Haemophilus (OTU 589)	*Haemophilus influenzae (OTU 5)*	0.651	<0.001
Haemophilus (OTU 536)	*Haemophilus influenzae (OTU 5)*	0.706	<0.001
Haemophilus haemolyticus (OTU 20)	*Haemophilus influenzae (OTU 5)*	0.543	<0.001
Fusobacterium (OTU 36)	*Haemophilus influenzae (OTU 5)*	0.002	0.978
Streptobacillus moniliformis (OTU 78)	*Haemophilus influenzae (OTU 5)*	0.073	0.391
Alloprevotella (OTU 29)	*Haemophilus influenzae (OTU 5)*	-0.011	0.897
Unassignable (OTU 32)	*Haemophilus influenzae (OTU 5)*	-0.061	0.473
Porphyromonas (OTU 26)	*Haemophilus influenzae (OTU 5)*	0.046	0.588
Porphyromonas catoniae (OTU 55)	*Haemophilus influenzae (OTU 5)*	0.000	0.997
Prevotella melaninogenica (OTU 16)	*Haemophilus influenzae (OTU 5)*	-0.018	0.835
Neisseria flavescens (OTU 23)	*Haemophilus influenzae (OTU 5)*	0.080	0.346
Moraxella (OTU 3)	*Haemophilus influenzae (OTU 5)*	-0.282	<0.001
Haemophilus parainfluenzae (OTU 31)	*Haemophilus influenzae (OTU 5)*	0.004	0.959

Neisseria (OTU 34)	*Haemophilus influenzae (OTU 5)*	-0.076	0.370
Rothia mucilaginosa (OTU 35)	*Haemophilus influenzae (OTU 5)*	-0.166	0.049
Corynebacterium (OTU 4)	*Haemophilus influenzae (OTU 5)*	-0.193	0.023
Streptococcus pneumoniae (OTU 1)	*Moraxella nonliquefaciens (OTU 571)*	-0.134	0.116
Staphylococcus (OTU 10)	*Moraxella nonliquefaciens (OTU 571)*	-0.098	0.249
Granulicatella (OTU 28)	*Moraxella nonliquefaciens (OTU 571)*	-0.124	0.143
Dolosigranulum pigrum (OTU 6)	*Moraxella nonliquefaciens (OTU 571)*	0.179	0.034
Streptococcus (OTU 9)	*Moraxella nonliquefaciens (OTU 571)*	-0.024	0.778
Veillonella (OTU 30)	*Moraxella nonliquefaciens (OTU 571)*	-0.073	0.393
Veillonella dispar (OTU 18)	*Moraxella nonliquefaciens (OTU 571)*	-0.214	0.011
Veillonella atypica (OTU 59)	*Moraxella nonliquefaciens (OTU 571)*	-0.052	0.542
Streptococcus (OTU 92)	*Moraxella nonliquefaciens (OTU 571)*	-0.100	0.238
Haemophilus (OTU 589)	*Moraxella nonliquefaciens (OTU 571)*	0.094	0.267
Haemophilus (OTU 536)	*Moraxella nonliquefaciens (OTU 571)*	-0.103	0.224
Haemophilus haemolyticus (OTU 20)	*Moraxella nonliquefaciens (OTU 571)*	-0.181	0.033
Fusobacterium (OTU 36)	*Moraxella nonliquefaciens (OTU 571)*	-0.135	0.113
Streptobacillus moniliformis (OTU 78)	*Moraxella nonliquefaciens (OTU 571)*	-0.137	0.107
Alloprevotella (OTU 29)	*Moraxella nonliquefaciens (OTU 571)*	-0.088	0.303
Unassignable (OTU 32)	*Moraxella nonliquefaciens (OTU 571)*	-0.126	0.137
Porphyromonas (OTU 26)	*Moraxella nonliquefaciens (OTU 571)*	-0.082	0.336
Porphyromonas catoniae (OTU 55)	*Moraxella nonliquefaciens (OTU 571)*	-0.044	0.603
Prevotella melaninogenica (OTU 16)	*Moraxella nonliquefaciens (OTU 571)*	-0.121	0.154
Neisseria flavescens (OTU 23)	*Moraxella nonliquefaciens (OTU 571)*	-0.032	0.709
Moraxella (OTU 3)	*Moraxella nonliquefaciens (OTU 571)*	0.436	<0.001
Haemophilus parainfluenzae (OTU 31)	*Moraxella nonliquefaciens (OTU 571)*	-0.109	0.198
Neisseria (OTU 34)	*Moraxella nonliquefaciens (OTU 571)*	0.040	0.637
Rothia mucilaginosa (OTU 35)	*Moraxella nonliquefaciens (OTU 571)*	-0.108	0.204
Corynebacterium (OTU 4)	*Moraxella nonliquefaciens (OTU 571)*	0.103	0.225
Haemophilus influenzae (OTU 5)	*Moraxella nonliquefaciens (OTU 571)*	-0.030	0.723
Streptococcus pneumoniae (OTU 1)	*Moraxella lincolnii (OTU 8)*	-0.173	0.041
Staphylococcus (OTU 10)	*Moraxella lincolnii (OTU 8)*	-0.164	0.053
Granulicatella (OTU 28)	*Moraxella lincolnii (OTU 8)*	-0.105	0.219
Dolosigranulum pigrum (OTU 6)	*Moraxella lincolnii (OTU 8)*	0.179	0.034

Streptococcus (OTU 9)	*Moraxella lincolnii (OTU 8)*	-0.033	0.703
Veillonella (OTU 30)	*Moraxella lincolnii (OTU 8)*	-0.023	0.789
Veillonella dispar (OTU 18)	*Moraxella lincolnii (OTU 8)*	-0.168	0.048
Veillonella atypica (OTU 59)	*Moraxella lincolnii (OTU 8)*	-0.120	0.158
Streptococcus (OTU 92)	*Moraxella lincolnii (OTU 8)*	-0.047	0.585
Haemophilus (OTU 589)	*Moraxella lincolnii (OTU 8)*	0.078	0.357
Haemophilus (OTU 536)	*Moraxella lincolnii (OTU 8)*	0.024	0.778
Haemophilus haemolyticus (OTU 20)	*Moraxella lincolnii (OTU 8)*	-0.099	0.245
Fusobacterium (OTU 36)	*Moraxella lincolnii (OTU 8)*	-0.059	0.489
Streptobacillus moniliformis (OTU 78)	*Moraxella lincolnii (OTU 8)*	-0.070	0.410
Alloprevotella (OTU 29)	*Moraxella lincolnii (OTU 8)*	-0.137	0.107
Unassignable (OTU 32)	*Moraxella lincolnii (OTU 8)*	-0.209	0.013
Porphyromonas (OTU 26)	*Moraxella lincolnii (OTU 8)*	-0.128	0.132
Porphyromonas catoniae (OTU 55)	*Moraxella lincolnii (OTU 8)*	-0.133	0.116
Prevotella melaninogenica (OTU 16)	*Moraxella lincolnii (OTU 8)*	-0.141	0.097
Neisseria flavescens (OTU 23)	*Moraxella lincolnii (OTU 8)*	-0.050	0.560
Moraxella (OTU 3)	*Moraxella lincolnii (OTU 8)*	0.285	<0.001
Haemophilus parainfluenzae (OTU 31)	*Moraxella lincolnii (OTU 8)*	-0.137	0.107
Neisseria (OTU 34)	*Moraxella lincolnii (OTU 8)*	-0.024	0.776
Rothia mucilaginosa (OTU 35)	*Moraxella lincolnii (OTU 8)*	-0.012	0.891
Corynebacterium (OTU 4)	*Moraxella lincolnii (OTU 8)*	0.178	0.035
Haemophilus influenzae (OTU 5)	*Moraxella lincolnii (OTU 8)*	0.108	0.202
Moraxella nonliquefaciens (OTU 571)	*Moraxella lincolnii (OTU 8)*	0.302	<0.001
Streptococcus pneumoniae (OTU 1)	*Granulicatella elegans (OTU 38)*	0.282	<0.001
Staphylococcus (OTU 10)	*Granulicatella elegans (OTU 38)*	0.138	0.103
Granulicatella (OTU 28)	*Granulicatella elegans (OTU 38)*	0.503	<0.001
Dolosigranulum pigrum (OTU 6)	*Granulicatella elegans (OTU 38)*	0.104	0.224
Streptococcus (OTU 9)	*Granulicatella elegans (OTU 38)*	0.343	<0.001
Veillonella (OTU 30)	*Granulicatella elegans (OTU 38)*	0.691	<0.001
Veillonella dispar (OTU 18)	*Granulicatella elegans (OTU 38)*	0.414	<0.001
Veillonella atypica (OTU 59)	*Granulicatella elegans (OTU 38)*	0.381	<0.001
Streptococcus (OTU 92)	*Granulicatella elegans (OTU 38)*	0.474	<0.001
Haemophilus (OTU 589)	*Granulicatella elegans (OTU 38)*	-0.024	0.781

Haemophilus (OTU 536)	*Granulicatella elegans (OTU 38)*	0.130	0.126
Haemophilus haemolyticus (OTU 20)	*Granulicatella elegans (OTU 38)*	0.368	<0.001
Fusobacterium (OTU 36)	*Granulicatella elegans (OTU 38)*	0.540	<0.001
Streptobacillus moniliformis (OTU 78)	*Granulicatella elegans (OTU 38)*	0.506	<0.001
Alloprevotella (OTU 29)	*Granulicatella elegans (OTU 38)*	0.670	<0.001
Unassignable (OTU 32)	*Granulicatella elegans (OTU 38)*	0.510	<0.001
Porphyromonas (OTU 26)	*Granulicatella elegans (OTU 38)*	0.701	<0.001
Porphyromonas catoniae (OTU 55)	*Granulicatella elegans (OTU 38)*	0.586	<0.001
Prevotella melaninogenica (OTU 16)	*Granulicatella elegans (OTU 38)*	0.485	<0.001
Neisseria flavescens (OTU 23)	*Granulicatella elegans (OTU 38)*	0.632	<0.001
Moraxella (OTU 3)	*Granulicatella elegans (OTU 38)*	-0.017	0.844
Haemophilus parainfluenzae (OTU 31)	*Granulicatella elegans (OTU 38)*	0.527	<0.001
Neisseria (OTU 34)	*Granulicatella elegans (OTU 38)*	0.593	<0.001
Rothia mucilaginosa (OTU 35)	*Granulicatella elegans (OTU 38)*	0.421	<0.001
Corynebacterium (OTU 4)	*Granulicatella elegans (OTU 38)*	0.025	0.769
Haemophilus influenzae (OTU 5)	*Granulicatella elegans (OTU 38)*	0.046	0.586
Moraxella nonliquefaciens (OTU 571)	*Granulicatella elegans (OTU 38)*	0.062	0.467
Moraxella lincolnii (OTU 8)	*Granulicatella elegans (OTU 38)*	0.001	0.987
Streptococcus pneumoniae (OTU 1)	*Gemella haemolysans (OTU 17)*	0.314	<0.001
Staphylococcus (OTU 10)	*Gemella haemolysans (OTU 17)*	0.320	<0.001
Granulicatella (OTU 28)	*Gemella haemolysans (OTU 17)*	0.490	<0.001
Dolosigranulum pigrum (OTU 6)	*Gemella haemolysans (OTU 17)*	0.047	0.580
Streptococcus (OTU 9)	*Gemella haemolysans (OTU 17)*	0.456	<0.001
Veillonella (OTU 30)	*Gemella haemolysans (OTU 17)*	0.687	<0.001
Veillonella dispar (OTU 18)	*Gemella haemolysans (OTU 17)*	0.524	<0.001
Veillonella atypica (OTU 59)	*Gemella haemolysans (OTU 17)*	0.345	<0.001
Streptococcus (OTU 92)	*Gemella haemolysans (OTU 17)*	0.600	<0.001
Haemophilus (OTU 589)	*Gemella haemolysans (OTU 17)*	-0.088	0.301
Haemophilus (OTU 536)	*Gemella haemolysans (OTU 17)*	0.132	0.121
Haemophilus haemolyticus (OTU 20)	*Gemella haemolysans (OTU 17)*	0.396	<0.001
Fusobacterium (OTU 36)	*Gemella haemolysans (OTU 17)*	0.542	<0.001
Streptobacillus moniliformis (OTU 78)	*Gemella haemolysans (OTU 17)*	0.438	<0.001
Alloprevotella (OTU 29)	*Gemella haemolysans (OTU 17)*	0.701	<0.001

Unassignable (OTU 32)	*Gemella haemolysans (OTU 17)*	0.582	<0.001
Porphyromonas (OTU 26)	*Gemella haemolysans (OTU 17)*	0.590	<0.001
Porphyromonas catoniae (OTU 55)	*Gemella haemolysans (OTU 17)*	0.619	<0.001
Prevotella melaninogenica (OTU 16)	*Gemella haemolysans (OTU 17)*	0.545	<0.001
Neisseria flavescens (OTU 23)	*Gemella haemolysans (OTU 17)*	0.581	<0.001
Moraxella (OTU 3)	*Gemella haemolysans (OTU 17)*	-0.201	0.017
Haemophilus parainfluenzae (OTU 31)	*Gemella haemolysans (OTU 17)*	0.548	<0.001
Neisseria (OTU 34)	*Gemella haemolysans (OTU 17)*	0.524	<0.001
Rothia mucilaginosa (OTU 35)	*Gemella haemolysans (OTU 17)*	0.539	<0.001
Corynebacterium (OTU 4)	*Gemella haemolysans (OTU 17)*	-0.023	0.787
Haemophilus influenzae (OTU 5)	*Gemella haemolysans (OTU 17)*	-0.048	0.576
Moraxella nonliquefaciens (OTU 571)	*Gemella haemolysans (OTU 17)*	-0.069	0.420
Moraxella lincolnii (OTU 8)	*Gemella haemolysans (OTU 17)*	-0.068	0.423
Granulicatella elegans (OTU 38)	*Gemella haemolysans (OTU 17)*	0.662	<0.001

Abbreviations. OTU: Operational Taxonomic Unit

Table S5. Bacterial correlations at the genus level. Spearman's correlation coefficients and associated *P*-values for correlations between most abundant genera detected in the nasopharyngeal samples from the children under study (median relative abundances >0.02%). All significant and non-significant bacterial interrelations are shown.

**Genus 1**	**Genus 2**	**Correlation coefficient**	***P*-value**
Corynebacterium	*Moraxella*	0.152	0.073
Corynebacterium	*Haemophilus*	-0.171	0.044
Corynebacterium	*Dolosigranulum*	0.751	<0.001
Corynebacterium	*Staphylococcus*	0.126	0.138
Corynebacterium	*Streptococcus*	-0.173	0.041
Dolosigranulum	*Staphylococcus*	0.286	<0.001
Dolosigranulum	*Streptococcus*	-0.136	0.108
Haemophilus	*Dolosigranulum*	-0.213	0.012
Haemophilus	*Staphylococcus*	-0.297	<0.001
Haemophilus	*Streptococcus*	-0.004	0.964
Moraxella	*Haemophilus*	-0.335	<0.001
Moraxella	*Dolosigranulum*	0.238	0.005
Moraxella	*Staphylococcus*	-0.043	0.618
Moraxella	*Streptococcus*	-0.166	0.050
Staphylococcus	*Streptococcus*	0.160	0.059
